# Quantitatively linking morphology and optical response of individual silver nanohedra†

**DOI:** 10.1039/d2nr02131e

**Published:** 2022-07-15

**Authors:** Yisu Wang, Zoltan Sztranyovszky, Attilio Zilli, Wiebke Albrecht, Sara Bals, Paola Borri, Wolfgang Langbein

**Affiliations:** School of Biosciences, Cardiff University Museum Avenue Cardiff CF10 3AX UK; School of Physics and Astronomy, Cardiff University The Parade Cardiff CF24 3AA UK langbeinww@cardiff.ac.uk; Department of Physics, Politecnico di Milano Piazza Leonardo da Vinci 32 20133 Milano Italy; EMAT and NANOlab Center of Excellence, University of Antwerp Groenenborgerlaan 171 B-2020 Antwerp Belgium

## Abstract

The optical response of metal nanoparticles is governed by plasmonic resonances, which are dictated by the particle morphology. A thorough understanding of the link between morphology and optical response requires quantitatively measuring optical and structural properties of the same particle. Here we present such a study, correlating electron tomography and optical micro-spectroscopy. The optical measurements determine the scattering and absorption cross-section spectra in absolute units, and electron tomography determines the 3D morphology. Numerical simulations of the spectra for the individual particle geometry, and the specific optical set-up used, allow for a quantitative comparison including the cross-section magnitude. Silver nanoparticles produced by photochemically driven colloidal synthesis, including decahedra, tetrahedra and bi-tetrahedra are investigated. A mismatch of measured and simulated spectra is found in some cases when assuming pure silver particles, which is explained by the presence of a few atomic layers of tarnish on the surface, not evident in electron tomography. The presented method tightens the link between particle morphology and optical response, supporting the predictive design of plasmonic nanomaterials.

## Abstract 2

A method linking three-dimensional morphology and quantitative optical scattering and absorption cross-sections of individual plasmonic nanoparticles is demonstrated, enabling a quantitative understanding of the morphology–optical response relation.

## Graphical abstract

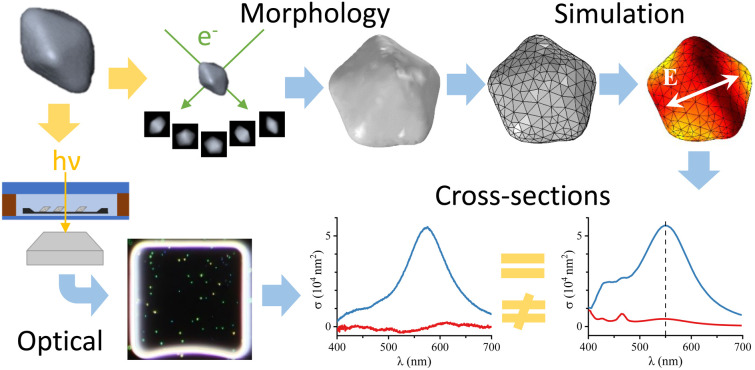

## 1 Introduction

Plasmonic nanoparticles (NPs) have optical properties which are controlled by their morphology. This enables a wide tuneability using a single material, such as silver or gold, just by size and shape control,^1^ including chirality and the associated chiro-optical response.^2^ The NP optical properties are described in terms of the cross sections for optical scattering (*σ*_sca_) and absorption (*σ*_abs_), which represent the strength of the NP–radiation interaction.^3^ While many experimental techniques have been developed to characterize the optical response at a single NP level,^4,5^ only few of these methods are able to quantify both optical cross sections in absolute units,^6^ or equivalently, the complex polarizability of the NP.^7^

Previous studies of correlative single-NP optical–electron microscopy using scattering spectra show the complex and sensitive dependence of the optical response on the morphology.^8^ Numerical modelling of the optical response based on a 3D reconstruction from electron tomography was shown in ref. 9, using discrete dipole approximation (DDA) simulations of a faceted gold NP, and for large irregular gold NPs simulated scattering spectra were compared with experiments.^10^ Furthermore, gold–silver core–shell NPs were investigated, either showing simulations for a given morphology^11^ or comparing simulations with measured scattering spectra as function of shell thickness.^12^ However, the above works did not attempt an accurate comparison of measured and simulated cross sections, and focussed on the spectral features instead. Over the past years, we have developed a measurement and data analysis method to retrieve accurately quantitative cross-section spectra.^13,14^ In ref. 15 we combined this method with standard projection transmission electron microscopy (TEM) to investigate silver cubes. The cube geometry results in NPs orientated such that one of the flat sides is attached to the TEM grid, so that the NP geometric parameters can be reasonably extracted from projection images. For more complex shapes, however, conventional TEM is insufficient, and electron tomography is needed.

In the present work, we study faceted silver NPs produced by photochemically driven colloidal synthesis,^16–18^ including decahedra, tetrahedra and bi-tetrahedra. Similar to their gold counterparts,^19,20^ their response is ruled by localized surface plasmon resonances. The chemical reactivity of silver surfaces makes these systems attractive for catalysis applications,^21,22^ but also provides a route to chemical surface modifications which can be difficult to identify in TEM images while significantly modifying the optical response.^23^ We find here that an accurate quantitative study of cross-section spectra correlating experiment with simulation can uncover such detail. The presented case study on the one hand assesses the level of accuracy that can be achieved by our cross-section measurement method, and on the other hand exemplifies the kind of fine information that can be extracted from quantitative cross-section spectroscopy. Ultimately such progress might enable to reliably extract the 3D morphology of metal NPs from optical measurements alone.

## 2 Materials and methods

**Fig. 1:**
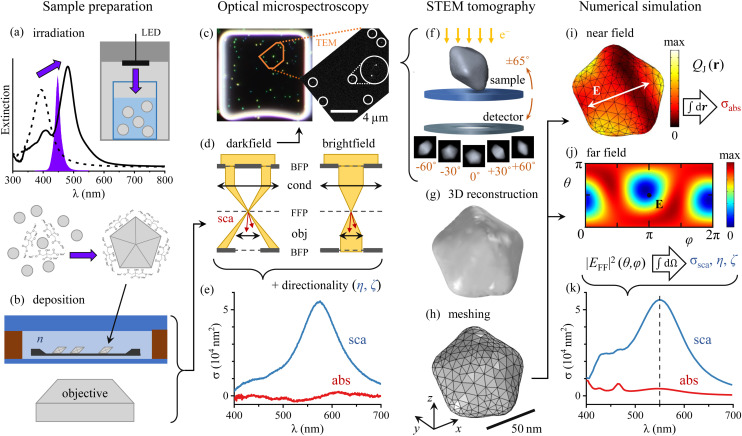
Schematic workflow as described in the text. (a) Photochemical formation of decahedra using blue LED illumination, monitored *via* the red-shift of the extinction from spherical seeds (dashed line) to decahedra (solid line). (b) Deposition of decahedra onto a TEM grid with SiO_2_ windows, index-matched by anisole immersion, and encapsulated by a glass slide and a coverslip. (c, d) Optical micro-spectroscopy in dark-field and bright-field configurations. BFP and FFP indicate, respectively, the back and front focal plane of the objective (obj) and condenser (cond) lens. (e) Measured single-decahedra scattering and absorption cross-section spectra in absolute units. (f) Correlative HAADF-STEM tomography through recognition of NP patterns as exemplified in (c). (g) 3D shape reconstruction from tomography. (h) Tetrahedral volume mesh used in numerical simulations. (i) Calculated spatial distribution of the Joule (resistive) heating. (j) Calculated far-field distribution of the scattering intensity. (k) Numerical simulations of cross-section spectra under experimental conditions. Panels e–k refer to the exemplary particle #20.

Let us present the workflow of the experiment summarized in Fig. 1. Silver decahedra NPs are fabricated with a plasmon-driven method adapting the protocols of Zheng *et al.*^17^ and Pietrobon and Kitaev.^16^ As shown in Fig. 1a, seeds grown by reduction of AgNO_3_ in aqueous solution are thought to aggregate to form decahedra under irradiation by a high power light-emitting diode (LED) centred at a 447 nm wavelength (violet spectrum in the graph). The formation of decahedra can be monitored *via* the progressive red shift of the extinction peak of the NP solution from spherical seeds (dashed line) to decahedra (solid line). Further details of the fabrication process and a kinetic study are reported in the ESI section S.I.†

As particle support for the correlative measurements we used a TEM grid (Ted Pella, 21530-10) composed of a 40 nm-thick SiO_2_ film (refractive index *n* = 1.46) supported by a 200 nm-thick Si_3_N_4_ film with 50 × 50 μm^2^ square windows, on a silicon substrate (one such window is the bright frame of Fig. 1c). The grid was washed using two repetitions of the sequence deionised water – acetone – anisole – ethanol, and then dried in air. The grid was held by a Teflon-coated stainless steel reverse-action tweezer throughout the functionalisation and washing process. The grid was incubated for 1 hour at 55 °C in 10 mL etching solution of 500 μL HCl (99%) diluted in 9.5 mL of 30% H_2_O_2_. The grid was then washed three times in water, followed by three times in ethanol. 200 μL of (3-aminopropyl) triethoxysilane (APTES) (Sigma Aldrich) was centrifuged at 20k RCF for 20 min to spin down any large debris. 100 μL of this APTES stock was then diluted in 9.9 mL ethanol (absolute, for HPLC, >99.8%, Sigma Aldrich) to obtain a 1% APTES solution, in which the grid was incubated for 1 hour. The grid was then washed three times in ethanol followed by three times in water. The resulting functionalised grid was dried in air at 55 °C for 30 min and stored at 4 °C for no longer than one month. The decahedra solution (9 μl of 0.25 optical density at 475 nm) was wet-cast (see ref. 15) onto the functionalised grid. The grid was subsequently washed by gently and repeatedly dipping in water, and then dipped in ethanol and dried.

To provide the NPs with a nearly homogeneous optical environment for the cross-section measurements, the TEM grid was sealed in anisole (*n* = 1.52) between a microscope slide (25 × 75 mm^2^, Menzel Gläser) and a coverslip (#1.5, 25 × 25 mm^2^, Menzel Gläser) using a 0.5 mm thick adhesive silicone spacer (Grace Bio-Labs 664507), with the TEM grid surface facing the coverlip side. We chose anisole rather than microscope immersion oil as it is volatile and evaporates without leaving residuals, enabling subsequent electron microscopy. This assembly is mounted onto an inverted optical microscope (Nikon, Eclipse Ti-U) with a 40× dry objective (Nikon MRD00405, CFI plan apochromat λ series) of 0.95 numerical aperture (NA) as depicted in Fig. 1b.

The procedure for the optical measurements and the quantitative analysis of the optical cross sections is largely the same we adopted in ref. 15. We therefore limit ourselves here to recapitulate the main steps performed and parameters used, while we refer the reader to the aforementioned work^15^ for an in-depth description. Single-particle microspectroscopy is performed by optically relaying the intermediate image plane created by the tube lens of the microscope onto the entrance slit of an imaging spectrometer (Horiba Jobin Yvon, iHR550) equipped with a ruled plane diffraction grating (Horiba, 51048) of 78 mm square size and 100 lines per mm. Spectra were acquired with a Peltier-cooled back-illuminated charge-coupled device (CCD) sensor (Andor, Newton DU-971N). The spectrometer images the entrance slit onto the sensor, allowing to use the zeroth order of the grating to provide an image of the sample to select a specific particle for spectroscopy. The entrance slit acts as a spatial filter in the horizontal direction (along the spectral dispersion), whereas in the vertical direction the binning of the CCD sensor itself is used to define a region of interest. Together these define a 1.0 × 1.0 μm^2^ square region centred on the NP of interest from which the signal is collected. The corrections required to account for this finite region of detection are described in section S.III of the ESI.†

Within the transillumination scheme adopted, we define two imaging modalities based on the angular range of the illumination, as illustrated in Fig. 1d. In the first one – a bright-field (BF) scheme – the illumination NA range is set to match the collection range (0–0.95) of the objective. In the second one – a dark-field (DF) scheme, the illumination range 1.06–1.34 NA is used, not overlapping with the collection range, so that only scattering is detected. As a result, scatterers such as NPs are visible as bright diffraction-limited spots on a dark background – see for example Fig. 1c (left). The two illumination ranges are defined by two corresponding 3D-printed apertures placed in the back focal plane (BFP) of the condenser lens (Nikon, T-C-HNAO, 1.34 NA oil-immersion) on a slider, which allows the reproducible switching between BF and DF required for an accurate correlation between transmitted and scattered light intensity.

The optical cross sections are defined as the power removed from the exciting beam per excitation intensity: *σ* = *P*/*I*_exc_. Thus, a careful referencing to the exciting intensity^24^ of the single-particle extinction and scattering spectra enables us to measure accurately the magnitude of the cross sections. Note that the BF extinction signal includes contributions of both absorption and scattering, which have to be unravelled based on the scattering-only DF signal. Such retrieval procedure is presented in ref. 13, and requires information on the directional properties of the scattering process. In the analysis this information is reduced to two parameters named *η* and *ζ*. *η* concerns the detection, and is the fraction of the total scattering collected by the objective. We note that *η* depends on the angular range of the illumination, such that *η*^BF^≠*η*^DF^; however, the difference is small for the decahedra, whose response is governed by the same dipolar mode under both BF and DF illumination. *ζ* concerns the excitation, and is the BF-to-DF ratio of the scattered power; it depends therefore on the relative intensity of the BF-to-DF illumination (which we characterised for our set-up as described in section S.IV of the ESI†), as well as on how much the resonant modes of the scatterer are excited under either illumination. In this work, *η* and *ζ* are computed numerically for each studied NP as described below. The details of the NP geometry for the cases studied here have a moderate effect, and therefore the values are rather similar for all NPs considered, see the ESI section S.V.† Following the quantitation procedure outlined above, we can measure cross-section spectra in absolute units, such as nm^2^ in Fig. 1e. Note that *σ*_sca_(*λ*) and *σ*_abs_(*λ*) refer to a given illumination and collection range. Specifically, in this work we measure *σ*^DF^_sca_ and *σ*^BF^_abs_, which differ^24^ from the cross sections under plane-wave excitation.

As illustrated by Fig. 1c, optical and electron microscopy images can be correlated through the recognition of a specific NP pattern. In the high-angle annular dark-field scanning TEM (HAADF-STEM) overview on the right, white circles highlight the NPs visible, and a distinctive dimer in the middle is shown magnified. We are thereby able to select the NPs characterised optically for HAADF-STEM tomography, wherein the sample is tilted across a wide angular range under the electron beam, as depicted in Fig. 1f, and the resulting stack of projection images is used to reconstruct the three-dimensional (3D) morphology of the NP. All electron tomography series were acquired using a FEI Tecnai Osiris electron microscope operated at 200 kV. The series are taken across the largest tilt range allowed by the TEM grids clearance – typically about ±65° – with a tilt increment of 3°. The 1k × 1k projection images are aligned to match the NP positions across each series using cross-correlation, and are then reconstructed using 15 iterations of the expectation–maximization reconstruction algorithm implemented in the ASTRA toolbox for MATLAB.^25,26^ The resulting reconstructions are down sampled by a factor 12 and segmented using the Otsu method to export them as .stl files, such as the one shown in Fig. 1g. This geometry is then meshed in COMSOL for numerical simulation purposes with a free tetrahedral volume mesh displayed in Fig. 1h. The influence of variations of this reconstruction procedure on the simulated cross-section spectra is investigated in subsection 3.1.

The optical response of the particles is computed in the frequency domain using COMSOL Multiphysics®, a commercial software implementing the finite-element method. In the model, the NP is defined as silver using the permittivity reported in ref. 27, immersed in a homogeneous medium of anisole (*n* = 1.52). We neglected the small index mismatch between the thin silica window (*n* = 1.46) and anisole and used a homogeneous medium instead of a multi-layered structure, therefore the model used here is equivalent to the one described in the ESI of our previous work ref. 15, with the slab thickness set to zero (*d* = 0 nm). This simplification allowed us to automate the importing and alignment of particle geometries from HAADF-STEM tomography into COMSOL. The stationary solution of Maxwell's equations under plane-wave (PW) excitation of given frequency, polarization, and propagation direction computed by COMSOL determines the spatial distribution of the electromagnetic field 
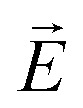
.

Let us now discuss how we derive the observables of interest (namely *σ*_abs_, *σ*_sca_, *η*, *ζ*) from this solution. Fig. 1i shows the spatial distribution of the Joule (resistive) heating 
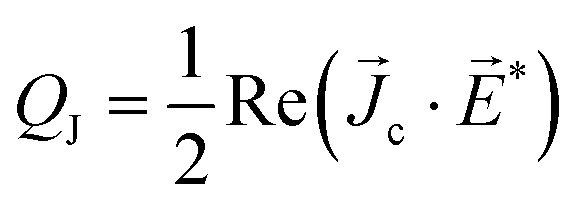
 where 
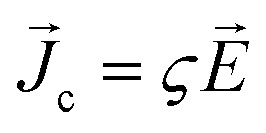
 is the conduction current in terms of the AC electrical conductivity *ς*. We integrate *Q*_J_ over the NP volume to compute the absorbed power *P*_abs_, and hence *σ*^PW^_abs_ = *P*_abs_/*I*_exc_ dividing by the excitation intensity *I*_exc_. The near-field solution can be projected to the far field *via* the far-field transform available in COMSOL, resulting in an angular distribution of the field 
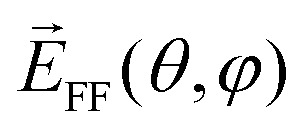
 such as the one shown in Fig. 1j.

A dipole-like emission pattern is seen, with the dipole oriented close to the *x* direction (identified by the polar angle *θ* = π/2 and the azimuth *φ* = 0, π, 2π) – albeit not precisely along it, due to a tilt of a long axis of the particle, along which its polarizability is maximized. The far-field Poynting vector 
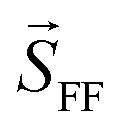
 (which is proportional to 
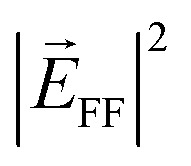
 plotted in Fig. 1j) can be integrated over the appropriate solid angle (4π or the objective acceptance) to compute the scattered power *P*_sca_ (and hence *σ*^PW^_sca_ = *P*_sca_/*I*_exc_) and the collected fraction of scattering *η*.

We emphasize that these values of *σ* and *η* are computed under PW excitation, which we have indicated with the PW superscript; in the experiment instead we use the incoherent illumination produced by a high-NA condenser, which is composed of a wide range of directions. To reproduce the measured *σ*^DF^_sca_ and *σ*^BF^_abs_ we therefore perform and average a large number of PW simulations sampling the directional range of illumination (either BF or DF), each direction being assigned an appropriate weight according to the angular dependence of the illumination intensity in our microscope, that we have characterised. The unpolarised illumination is reproduced by averaging for each direction the results of two PW simulations with orthogonal excitation polarisation, namely p and s with respect to the imaged sample plane. An analogous directional averaging is applied to compute the scattering parameters (*η*^BF^, *η*^DF^, and *ζ*) appropriate to the experimental angular ranges of illumination and detection. The mathematical details of such procedure are given in ref. 15 section S.IV to S.VI.

This averaging results in the *σ*^DF^_sca_ and *σ*^BF^_abs_ spectra, which are shown in Fig. 1k, and are quantitatively simulating the experimental ones in Fig. 1e. From here on we drop the DF and BF superscript of the cross sections for simplicity. In the next section we will compare in detail the experimental and simulated cross sections, focussing on their differences to identify additional aspects of the system beyond its measured geometry yet to be included in the model. In this manner, the comparison can bring about additional knowledge on the system – such as the presence of surface layers or variations of the metal permittivity.

## 3 Results and discussion

**Fig. 2:**
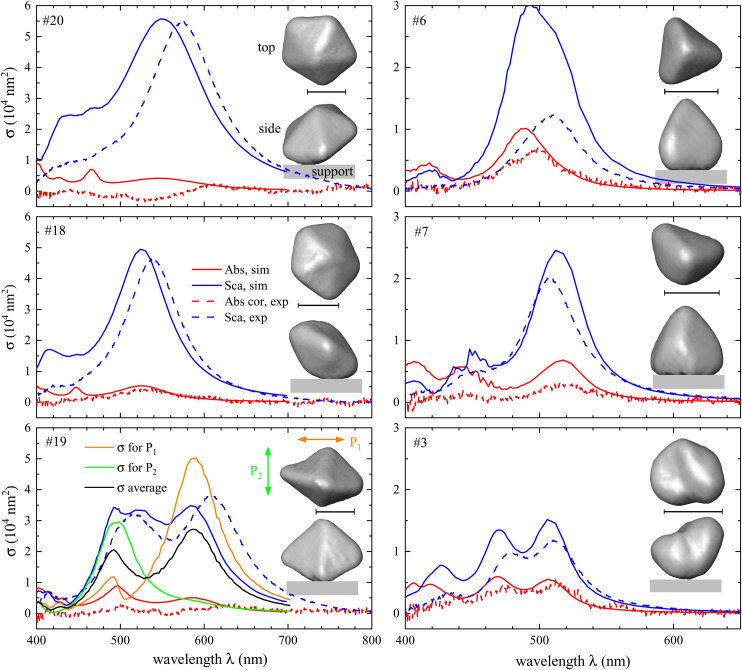
Measured (dashed lines) and simulated (solid lines) scattering (blue) and absorption (red) cross-section spectra of 6 selected particles as labelled, along with HAADF-STEM tomography surface views from the top and side. The scale bar is 40 nm. For particle #19, we show additionally the simulated scattering cross section for normal incidence for linear polarizations along (orange line) and across (green line) the long axis of the particle, as well as their average (black line).

**Fig. 3:**
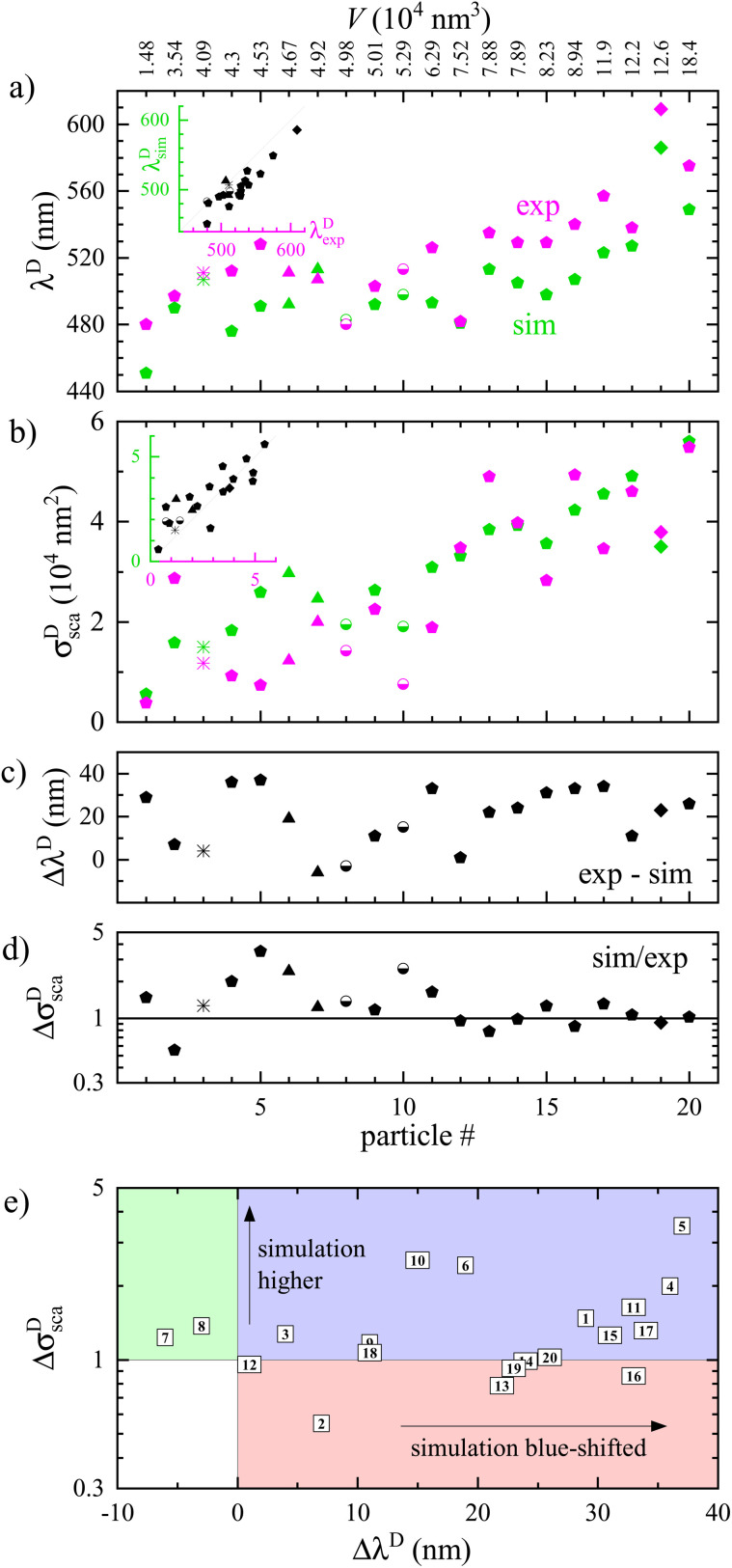
Comparison of measured and simulated properties of the dipole peak in the scattering cross-section spectra for all investigated particles. (a) Position of the peak *λ*^D^_sca_. For particles with multiple peaks, such as #19 or #3, the longer wavelength peak is shown. The symbols are indicative of the particle shape (see insets in Fig. 2 and ESI section S.V.†): #6 & #7 are tetrahedra, #8 & #10 are half spheres, #19 is a bitetrahedron, #3 is not well defined, the rest are decahedra. The inset shows simulated *versus* measured positions. (b) Amplitude of the peak. The inset shows simulated *versus* measured amplitudes. (c) Difference between the simulated and experimental peak position. (d) Ratio between simulated and experimental peak amplitude. (e) Peak amplitude ratio *versus* position difference.

Twenty particles were measured in total, which we numbered with increasing volume *V*. Fig. 2 shows the measured and simulated cross-section spectra for six selected particles representing the range of shapes and sizes, along with the top and side view of their 3D reconstructions. The data for the remainder of the particles are shown in the ESI section S.V.† Animated 3D renderings of the NP reconstructions are shown in the ESI section S.VI.C.† The top view shows the particle as seen along the illumination axis, indicating the main plane of excitation polarizations, even though due to the high NA also axial polarization is present, more markedly for the DF illumination. While the fabrication method was developed to produce decahedra (such as particles #20 and #18), other shapes are present, such as tetrahedra (#6 and #7), or a bi-tetrahedron (#19). The particles range in sizes, as summarized in Fig. 3. The decahedra and tetrahedra show a single pronounced peak in the scattering cross section, at a wavelength between 500 and 550 nm. The more elongated particles, #19 and #3, show two distinct peaks, which are dipolar modes with polarisations along the longer or shorter axis, centred at longer or shorter wavelengths, respectively. COMSOL simulations of the scattering cross section of particle #19 under normal-incidence plane-wave illumination polarized along the shorter and longer axis (green and orange lines, respectively) confirm this attribution. For most particles we find a reasonable agreement in the lineshape and magnitude of the scattering cross-section peak around the dipolar resonance, though the wavelength position shows a systematic blue shift of the simulated data relative to the measured one. The measured absorption spectra show regions of negative values, which is not expected, as it implies a net power emission by the particle. The absorption is determined as difference between extinction and scattering, using a range of numerically calculated and experimentally measured parameters, as mentioned in section 2. With the scattering dominating for most particles, the resulting small difference is affected by systematic errors in the measured extinction and scattering. These considerations and the wavelength dependence of the analysis parameters are discussed in more detail in the ESI section S.V.†

To correlate the results of experiment and simulations across all particles measured, we compare key spectral features in Fig. 3. The position of the dipolar scattering peak (panel a) shows a redshift with increasing particle index and thus particle volume, and the amplitude of the peak (b) increases with volume, both of these effects are generally well known and understood in literature.^18,28^ The quantitative comparison between measurements and simulations shows a remarkable agreement, considering that no adjustable parameters have been used. The difference between simulated and measured peak positions can be seen in the inset, and separately in (c). We find good correlation, with most particles showing a red shift of the measurement relative to the simulation by a few tens of nanometres. This finding is reminiscent of the shift observed in experiments with silver cubes.^15^ The relative deviation between simulated and measured peak magnitude (see panel d) shows a significant fluctuation, mostly with the simulation being higher, though the deviation decreases for large particles. Generally, the signal-to-noise ratio in the HAADF-STEM projection images is smaller for smaller particles, allowing for a larger relative error. In addition, the finite angular range used for the tomography reconstructions gives rise to a so-called missing wedge artefact, a result of a lack of information along certain directions. This can lead to systematic errors depending on the particle morphology, which could cause particle to particle fluctuations. On the optical measurement side, smaller cross sections are more affected by noise due to diffuse background scattering. However, the noise level is typically not significant in the present data, as can be seen in the scattering spectra shown in Fig. 2. On the other hand, the absorption displays a better agreement for small particles, as can be seen in the ESI section S.V.† This is due to the response of large particles being dominated by scattering, and the systematic error in the quantification of the absorption being proportional to the scattering, as previously discussed.

In Fig. 3e the particles are shown in a plane spanned by the ratio in amplitude and the difference in peak position between the measured and simulated data, to facilitate identifying and categorizing the possible sources for the discrepancy. The area shaded in red corresponds to both a blue shift and a decrease in amplitude of the simulated scattering dipole peak compared to the experimental one. It is known that rounding the edges of the particle causes a blue shift and a decrease of the magnitude of the plasmonic peaks. For example, it was observed for silver prisms,^29^ silver cubes,^15,30^ and gold decahedra.^31^ We note that the samples were shipped from the optical experiment at Cardiff to the electron tomography at Antwerp in nitrogen atmosphere in a sealed container at room temperature, providing up to 4 days during which such rounding might have developed.^32^ In the area shaded in blue the simulated peak is also blue shifted, but the simulated amplitude is higher than the experimental one. Based on our previous work^15^ this is likely due to a surface layer forming on the particles. An increased damping in the permittivity can also lead to a decrease of the scattering cross section, as we shall discuss in subsection 3.2 below. The green area corresponds to a red shift of the simulated spectra with respect to the experimental ones, with an increase in amplitude. The two particles in this area show rather small deviations, within the accuracy of determining the values.

Below we investigate some of these potential sources of deviation in more detail on two selected particles. As the cross-section simulations taking into account the wide NA range of the microscope illumination are computationally expensive, we increased the sampling step size of the illumination direction from 0.21 NA to 0.3 NA, reducing the simulation time by a factor of two, while affecting the cross-section spectra by less than a few percent.

### 3.1 Geometry reconstruction accuracy

The measured NP morphology dictates the simulated optical cross sections, and thus should be as accurate as possible. In our analysis pipeline, the reconstruction of the electron tomography depends on analysis parameters which influence the resulting morphology. As mentioned earlier, electron tomography suffers from the missing wedge artefact, which leads to a lack of information along certain directions, and we found that the resulting morphology slightly depended on the number of iterations in the reconstruction process. One can also include pre-processing of the data such as smoothing procedures. In addition, to achieve a reasonable simulation time, the NP morphology needs to be meshed with an acceptable number of elements, which depends on the computational power available and the accuracy required. In this section we discuss the influence of these points on the reconstructed morphology and simulated spectra.

We call R1 the meshed reconstructions used in Fig. 2 and 3, which employed 15 iterations of the expectation–maximization reconstruction algorithm and a downsampling factor of *N* = 12. Downsampling by a factor *N* bins together pixels in an *N* × *N* × *N* volume, so reduces the number of elements defining the NP's surface by a factor of *N*^2^. The exact number of facets depended on the NP, but in general for R1 the NP's surface geometry consisted of a few thousand faces. In the reconstruction procedure R2, we smoothed the input projection images with a pixel radius of 3 prior to the iterations to improve the signal-to-noise ratio, and reduced *N* to 4, which increased the number of surface elements to tens of thousands. For reconstruction R3, we furthermore increased the iterations to 100 and reduced *N* to 1, which increased the number of surface elements to hundreds of thousands.

For the large number of surface elements resulting from R2 and R3, COMSOL was unable to reliably import the geometry and construct usable particle models. To circumvent this problem we reduced the number of surface elements to approximately 1000 before importing. This did not cause a significant loss of accuracy: we observed typically around 5 nm blue shift and 1% increase in amplitude (see ESI section S.VI.† for details about the procedure and the effects). We note that the mesh on which COMSOL solves the scattering problem is usually even coarser. This mesh was determined by investigating the convergence of the simulated scattering cross-section amplitude at the dipole peak *versus* the mesh size, as described in the ESI of ref. 15 – we choose the size of NP mesh elements so that the calculated dipole resonance scattering amplitude is within 1% from the converged value, yielding about 500–1000 surface elements on the NP.

**Fig. 4:**
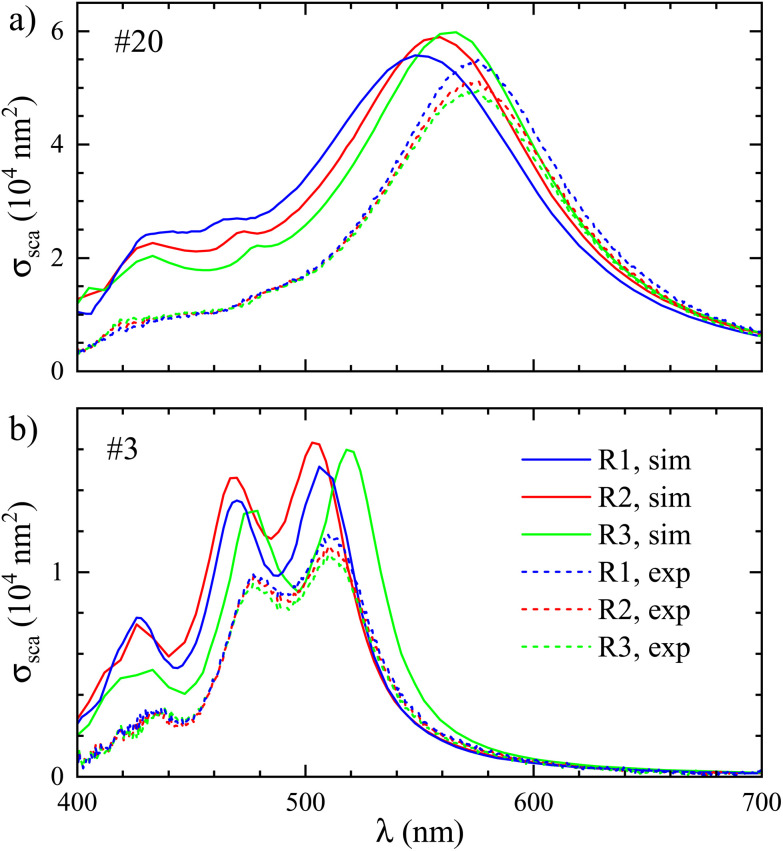
Simulated and measured scattering cross-section spectra for particle #20 (a) and #3 (b) using different tomography reconstruction procedures R1 to R3 as labelled (see text).

The reconstructions R2 and R3 resulted in a slightly altered geometry that was hardly discernable visually on the COMSOL mesh, so that we look here at the calculated volume and surface area changes (see ESI Table S1†), and the effect on the cross-section spectra, as shown in Fig. 4 (see ESI Fig. S14† for more examples). For particle #20, the volume is *V* = (18.4, 18.2, 17.4) × 10^4^ nm^3^ for (R1, R2, R3), and the volume to surface ratios are *V*/*S* = (10.8, 10.6, 10.4) nm. For particle #3 the volumes are *V* = (4.09, 4.29, 4.10) × 10^4^ nm^3^ and the volume to surface ratios are *V*/*S* = (6.65, 6.79, 6.5) nm.

For the larger particle (#20 shown in Fig. 4a), the reconstructions have little influence on the simulation results. Despite the decreasing volume, we observed a small red shift and increase in scattering cross section for R2 and R3. Noting that these reconstructions create less smoothing of morphological features, the red shift can be related to a sharpening of the geometry. For the smaller particle (#3 shown in Fig. 4b), R2 and R3 create different effects. For R2 we observe a small blue shift and a small increase in amplitude. The blue shift could result from remeshing, as mentioned before. The increase of the scattering amplitude is consistent with the increase in the volume. For R3 instead, we observe a red shift and slight redistribution of amplitude between the two peaks is seen. We attribute this to a sharpening of morphological features in the missing wedge region due to the higher number of iterations in the reconstruction algorithm. The slight increase in the splitting of the two peaks also suggests a small increase in aspect ratio. The modified simulated cross sections result in modified analysis parameters (*η*, *ζ*) which in turn modify the measured cross sections slightly, as shown by the dashed lines.

The results discussed in this section are indicative of the uncertainty originating from the reconstruction. For the following simulations we chose to use R2, having a slightly improved signal-to-noise ratio compared to R1 due to the additional smoothing of the input projections, but avoiding R3 where the high number of iterations may lead to a roughening of the morphology by an overfitting of noise in the expectation–maximization algorithm.

### 3.2 Modification of the permittivity

It is well known that the permittivity of a metal measured by ellipsometry on a planar surface can require a modification for NPs due to the reduced mean free path of the electrons.^33^ We accordingly model the effect of additional damping (combining the surface damping, the so-called chemical interface damping, and crystal defects) on the Ag permittivity *ε*_exp_(*ω*) measured by ellipsometry on a planar surface of polycrystalline Ag films^27^ as function of the angular frequency *ω* = 2π*c*/*λ*, with the speed of light *c* and the wavelength *λ*. We first fit *ε*_exp_(*ω*) in the wavelength range between 400 nm and 700 nm, avoiding the Ag interband transitions at shorter wavelengths, with a Drude model, *ε*(*ω*, *γ*) = *ε*_∞_ − *ω*_p_^2^/(*ω*^2^ + i*ωγ*), as detailed in the ESI section S.VII.,† where *ω*_p_ is the plasma frequency and *γ* is the damping. Then, we increase the damping by the term^33–36^*gv*_F_/*R*, where *v*_F_ is the Fermi velocity, *R* is the effective radius, and *g* is a scaling factor. We use the radius *R* calculated from the particle volume *V* assuming a spherical shape, 
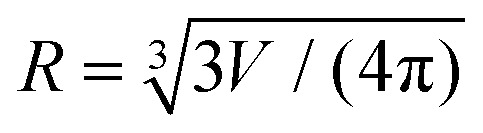
, resulting in *R* = 35.2 nm for particle #20 and *R* = 21.7 nm for particle #3. Finally, we add the permittivity change due to the increased damping to the measured permittivity data set, resulting in the modified permittivity *ε*_m_(*ω*) = *ε*_exp_(*ω*) + *ε*(*ω*, *γ* + *gv*_F_/*R*) − *ε*(*ω*, *γ*) to be used in the simulation.

**Fig. 5:**
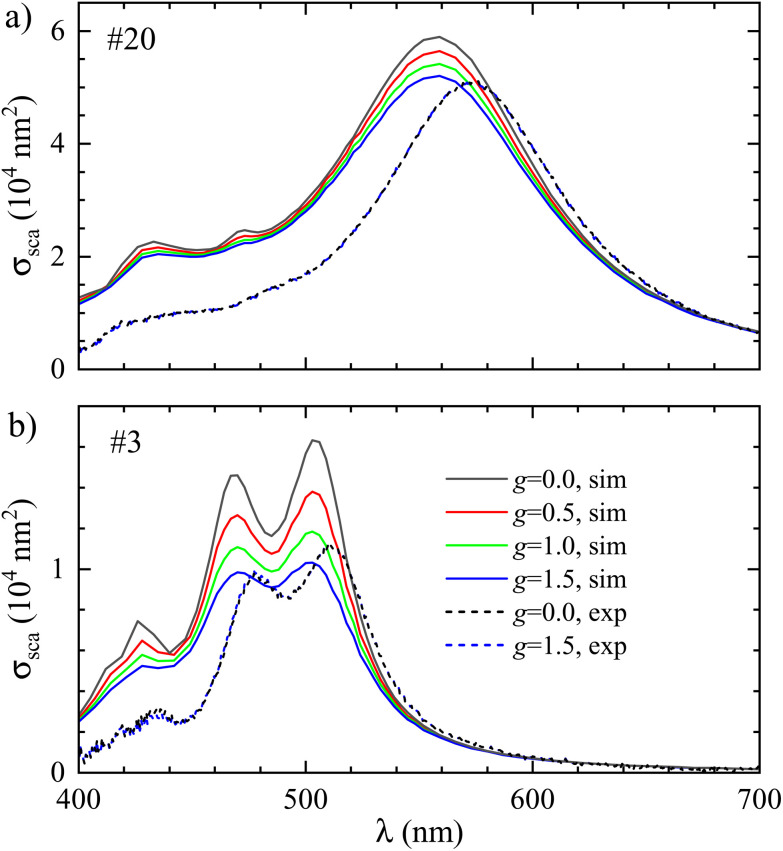
Same as Fig. 4, but for increasing surface scattering *gv*_F_/*R* in the Drude damping of the Ag permittivity.

The effect of the increased damping on the cross-section spectra is shown in Fig. 5. The scattering cross section decreases with increasing *g* from 0 to 1.5 (a typical range reported previously^36^), together with a broadening of the peaks, while the absorption cross section increases (see ESI Fig. S17†). The measured cross section does not change notably with *g*, showing that the analysis parameters (*η*^BF^, *η*^DF^, *ζ*) are not significantly affected by the additional damping.

### 3.3 Addition of a tarnish layer

While it might be possible that changing both reconstruction procedure and damping could produce a *σ*_sca_ matching the measurements, we did not find a reconstruction that would consistently move the spectra of all particles enough that a further permittivity change could explain the remaining discrepancy. Therefore we consider here another deviation of the particle description in the model from reality, given by an atomically thin chemical surface modification, which is not expected to be visible in the electron tomography for the imaging settings used. Such a layer, which can form on silver (as opposed to gold) due to its reactivity, is most likely sulfide or oxide.^37,38^ Both compounds have a high refractive index and also absorption, causing a red shift and a decrease in scattering magnitude,^15,39^ mimicking the observed mismatch between simulation and measurement for the majority of the particles.

More discussion and data regarding the possible origin and experimental evidence and of such layers, including energy-dispersive X-ray spectroscopy, is given in the ESI section S.II.†

To model such layers in COMSOL we used the following approach: starting from the surface mesh of the particle, we modeled a surface layer by isotropically scaling down the mesh, while fixing its center of mass, to define a Ag core of volume *V*_c_, with the remaining space in the original volume *V*_s_ providing the shell. The resulting average shell thickness *h* is taken as *h* = 2(*V*_s_ − *V*_c_)/(*A*_s_ + *A*_c_), where *A*_s_ and *A*_c_ are the surface areas before and after the scaling, respectively. Since sulfur is typically more reactive with Ag than oxygen, the wavelength-dependent permittivity of the shell was set to the one of silver sulfide.^40^

**Fig. 6:**
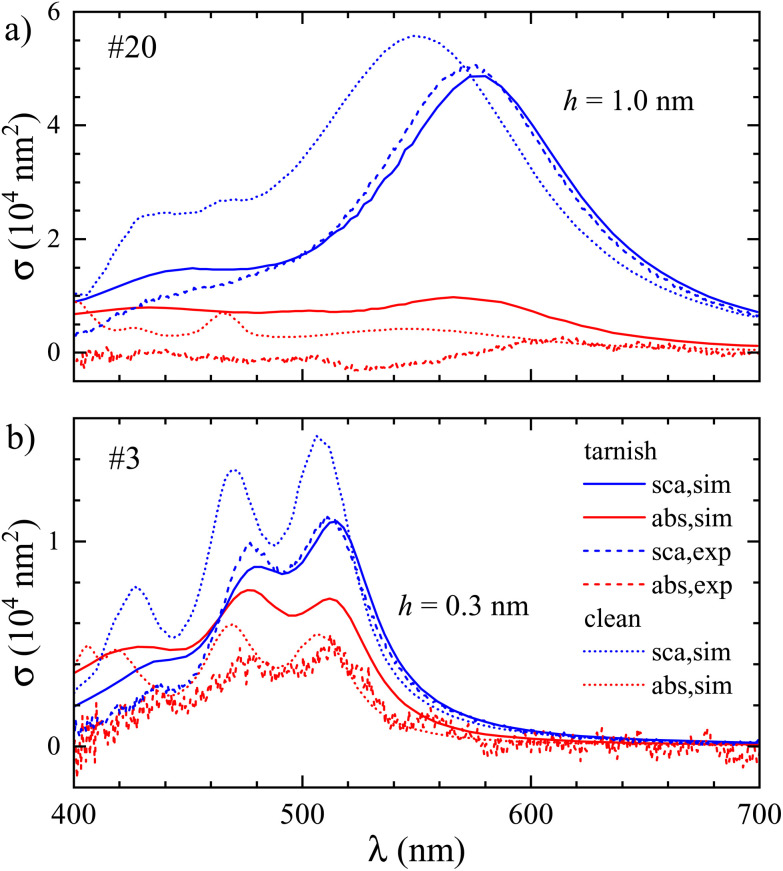
Same as Fig. 4, but for the addition of a silver sulfide (Ag_2_S) tarnish layer of thickness *h*, and additionally showing the absorption cross section.

For particle #20 we scaled down the mesh by a factor of 0.97, creating a layer of thickness *h* = 1.0 nm. For particle #3 we used a scaling factor of 0.985, yielding *h* = 0.3 nm. These shell thicknesses yield a good agreement between simulated and measured scattering cross-section spectra as shown in Fig. 6. For the absorption cross-section spectra, which are increased by the tarnish, some mismatch remains. We show in the ESI section S.VIII.† that assuming silver oxide instead of silver sulfide, a similar effect on the cross sections is found for a slightly larger thickness. Importantly, we note that a tarnish layer can have a much more complex morphology than assumed here, and can also contain a mixture of sulfide, oxide, and even other compounds such as FeS. A residual mismatch is therefore expected considering the simple tarnish model employed. We emphasize that for some of the NPs (*e.g.* #12, see ESI Fig. S10†) there is a good agreement between measured and simulated spectra within the expected uncertainty from the shape reconstruction (see ESI Fig. S14†), without adjustable parameters, indicating that the formation of a tarnish layer varies between particles even within the same preparation and TEM grid.

## 4 Conclusions

We have used the pipeline for correlative and quantitative optical and structural electron microscopy characterization that we have recently developed^15^ to study individual silver nanohedra synthesized by photochemistry. Importantly, we extended the method to include electron tomography to determine the volumetric shape of the particles accurately, and used the resulting morphology and orientation for simulations of the quantitative optical cross-section spectra, for a fitting-parameter free quantitative comparison with the measured spectra. This is the first study of this type, combining fully quantitative optical cross-section measurements with correlative electron tomography determining quantitatively the 3D particle morphology and orientation, and corresponding quantitative simulations.

While generally a good agreement of simulated and measured cross sections is found, quantitative differences are revealed. Specifically a red shift of the measurements compared to the simulations by a few percent, mostly for the larger particles, and a difference in magnitude, mostly a reduction for the smaller particles. To understand the origin of the deviations, the influence of three aspects was investigated. (i) The tomographic reconstruction method was examined, showing resulting morphology variations mostly for the smallest particles investigated. (ii) The addition of a realistic surface damping in the permittivity resulted in only slightly modified spectra. (iii) Adding a thin surface layer of tarnish, here modelled as silver sulfide, brought about, for realistic thicknesses in the 1 nm range, a match within the expected systematic errors. Let us emphasize that such conclusions would have been less stringent without the information on the cross-section magnitude. For instance, the red shift of the measured spectra can be explained both in terms of the geometry being more sharp, within the reconstruction accuracy, and by the tarnish layer; but only the latter hypothesis is in agreement with the measured cross-section magnitudes.

The accuracy of the method can be improved going forward. For example, one could add polarization-dependent measurements and simulations, using linearly, radially, and azimuthally polarised light, where the latter has the advantage of only in-plane polarized excitation for both BF and DF, thus exciting the same resonances. Furthermore, the slight angular dependence of the objective transmission could be calibrated and taken into account. To avoid the formation of a tarnish layer, a similar study on gold nanohedra could be envisaged, allowing to isolate the accuracy of geometry and permittivity.

This work and the adoption of the developed methodology paves the way towards an accurate quantitative understanding and verification of the morphology–optical response relation in plasmonic nanoparticles, especially for particles with complex shapes, which are important building blocks for next-generation devices.

## Author contributions

Y.W., P.B., and W.L. developed the workflow for correlative electron and optical microscopy. A.Z., P.B., and W.L. developed the quantitative optical microspectroscopy technique. Y.W. performed the sample preparation and optical microspectroscopy. A.Z., Z.S., P.B., and W.L. developed the numerical model and methods. Z.S. and A.Z. performed the numerical simulations. W.A. and S.B. performed electron tomography and analysis. A.Z., Z.S., and W.L. wrote the initial draft. All authors took part in interpreting the data and writing the manuscript.

CRediT: Conceptualization W.L., P.B., W.A.; data curation Y.W., Z.S., A.Z., W.A., W.L.; formal analysis Y.W., Z.S., A.Z., W.A., W.L.; funding acquisition W.A., S.B., P.B., W.L.; investigation Y.W., Z.S., W.A.; methodology Y.W., Z.S., A.Z., W.A., P.B., W.L.; project administration W.A., S.B., P.B., W.L.; resources S.B., P.B., W.L.; software Z.S., A.Z., W.L.; supervision A.Z., S.B., P.B., W.L.; validation Y.W., Z.S., A.Z., W.A.; visualization Y.W., Z.S., A.Z., W.A., W.L.; writing – original draft Y.W., Z.S., A.Z., W.A., W.L.; writing – review & editing Y.W., Z.S., A.Z., W.A., S.B., P.B., W.L.

## Data availability

Information about the data created during this research, including how to access it, is available on the Cardiff University data archive at https://doi.org/10.17035/d.2022.0176556287.

## Conflicts of interest

The authors declare no conflicts of interest.

## Supplementary Material

NR-014-D2NR02131E-s001

NR-014-D2NR02131E-s002

NR-014-D2NR02131E-s003

NR-014-D2NR02131E-s004

NR-014-D2NR02131E-s005

NR-014-D2NR02131E-s006

NR-014-D2NR02131E-s007

NR-014-D2NR02131E-s008

NR-014-D2NR02131E-s009

NR-014-D2NR02131E-s010

NR-014-D2NR02131E-s011

NR-014-D2NR02131E-s012

NR-014-D2NR02131E-s013

NR-014-D2NR02131E-s014

NR-014-D2NR02131E-s015

NR-014-D2NR02131E-s016

NR-014-D2NR02131E-s017

NR-014-D2NR02131E-s018

NR-014-D2NR02131E-s019

NR-014-D2NR02131E-s020

NR-014-D2NR02131E-s021
