# Supplementary material for: Quantitatively linking morphology and optical response of individual silver nanohedra
Source: Nanoscale. 2022 Jul 15;14(30):11028–37. doi: 10.1039/d2nr02131e (PMC9351607; doi:10.1039/d2nr02131e)
Supplement: NR-014-D2NR02131E-s001 [file NR-014-D2NR02131E-s001.pdf]

Electronic Supplementary Material (ESI) for Nanoscale.
This journal is © The Royal Society of Chemistry 2022

Electronic supplementary information to: Quantitatively linking morphology and optical response of individual silver nanohedra

Yisu Wang,[1,*] Zoltan Sztranyovszky,[2,*] Attilio Zilli,[1,3] Wiebke Albrecht,[4,†] Sara Bals,[4] Paola Borri,[1] and Wolfgang Langbein[2,‡]

[1]*School of Biosciences, Cardiff University – Museum Avenue, Cardiff CF10 3AX, UK*
[2]*School of Physics and Astronomy, Cardiff University – The Parade, Cardiff CF24 3AA, UK*
[3]*Department of Physics, Politecnico di Milano – Piazza Leonardo da Vinci 32, 20133 Milano, Italy*
[4]*EMAT and NANOlab Center of Excellence, University of Antwerp – Groenenborgerlaan 171, B-2020 Antwerp, Belgium*

(Dated: July 20, 2022)

[*] These authors contributed in equal measure to this work.
[†] Present address:
Department of Sustainable Energy Materials, AMOLF, Science Park 104, 1098 XG Amsterdam, The Netherlands.
[‡] langbeinww@cardiff.ac.uk

S.I. SAMPLE FABRICATION

The fabrication procedure is based on Ref. [S1]. The seeds were fabricated by reduction of silver nitrate ($AgNO_3$) in aqueous solution. 8 mL seed solution was prepared by mixing 0.5 µM silver nitrate (Sigma Aldrich), 6.25 µM polyvinylpyrrolidone (PVP) of molecular weight 10k (Sigma Aldrich), 3 mM trisodium citrate ($Na_3C_6H_5O_7$) (Sigma Aldrich) and 0.65 µM sodium hydroborate ($NaBH_4$) (Sigma Aldrich) with vigorous stir for 3 mins at room temperature until the color of the solution turned into light yellow. The seed solution, placed in a glass vial (Fisherbrand, type 1 class A borosilicate glass) and covered with a glass coverslip (Agar Scientific #1.5) was then irradiated for 7 hours at room temperature via a royal-blue (447 nm) LUXEON Rebel ES LED with a measured optical power of 710 mW in a home-built chamber with a cylindrical inner volume of 53 mm diameter and 95 mm height, made of aluminium. The inner surface of the chamber was painted first with a white primer (Starglow Universal Primer, Glowtec, UK) then by a reflective varnish (Starglow Clear Reflective Paint, Glowtec, UK) to achieve a high diffuse reflectivity ($> 95\%$) improving the intensity and homogeneity of the irradiation. The product solution after irradiation had an orange color, and was then purified via a 2-step centrifugation to minimise the aggregation in the pellet: a first step at 500 relative centrifugal force (RCF) for 20 min (to remove the small silver crystals) was followed by a second step at 1500 RCF for 20 min. After each step, the supernatant was removed and the pellet was resuspended in 0.1% PVP with 2 mM trisodium citrate solution. The product solution was stable (seen by a stable colour) in the fridge at 4°C for several months. All nanoparticles (NPs) analysed were synthesised no more than two days two before optical imaging. Conventional high resolution transmission electron microscopy (HRTEM) images of the purified solution were acquired on a JEOL JEM-1011 microscope equipped with a thermionic gun at 100 kV accelerating voltage. Samples were prepared by drop-casting NP suspensions onto carbon film-coated 200 mesh copper grids. Fig. S1 shows the fabricated NPs, which are dominantly decahedra, but also include triangular plates, bipyramids, and other shapes.

A preliminary kinetic study shown in Fig. S2 was performed to determine the formation times. The observed UV–Vis spectral evolution of the photochemical growth is consistent with data previously published in Ref. [S2]. A progressive rise of a plasmonic peak at 480 nm is observed, which is characteristic of Ag decahedra in aqueous solution.

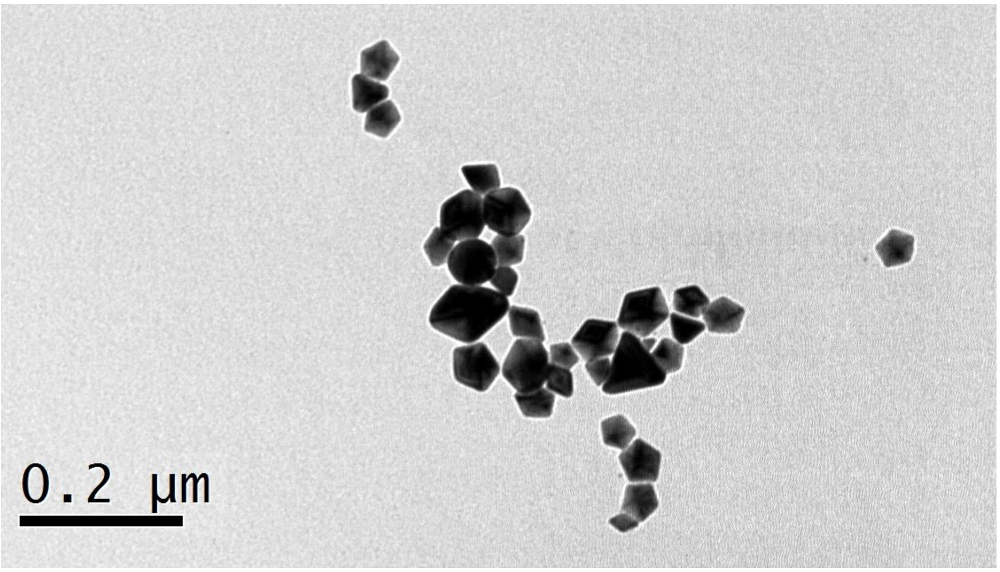

Figure S1. HR-TEM images (JEOL JEM-1011) of the drop-casted purified product solution.

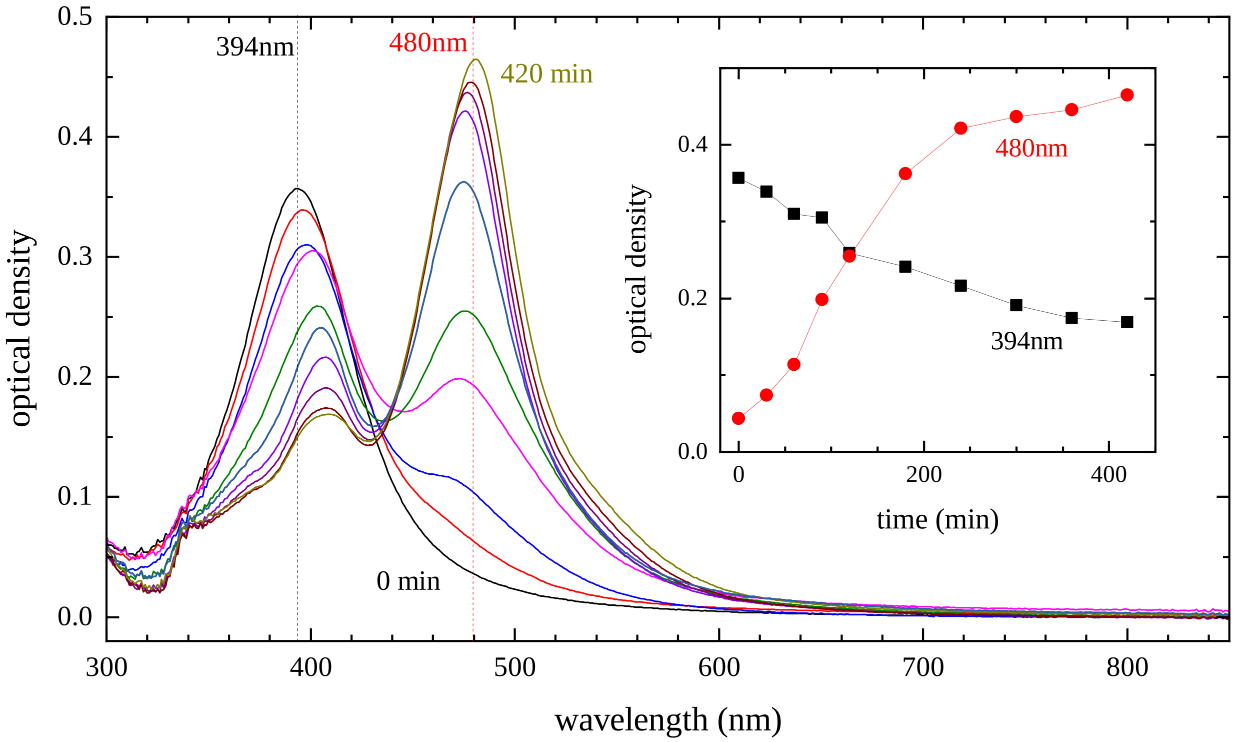

Figure S2. Kinetic study of decahedra formation under 455 nm LED irradiation: 7 mL of seed solution (optical density 3.5 at 400 nm) was irradiated with a LED (ThorLabs M455L3-C1) of 455 nm wavelength and 500 mW power with the sample and LED output in close proximity enclosed in aluminium foil. The irradiation was paused at different time points, and 50 μL of the reaction solution was sampled and diluted (1 in 10) for UV–Vis spectroscopy. Extinction spectra recorded using a Varian Cary 3000 UV–Vis spectrophotometer for various irradiation times are shown. The inset shows the measured optical density at 394 nm and 490 nm as a function of irradiation time.

S.II. SULFIDISATION

In the main text we discuss the influence of a thin Ag_2S tarnish layer, leading to a better agreement between simulated and measured optical cross-sections. Such a layer can form due to exposure to trace amounts of sulfur, either in the surrounding atmosphere (H_2S for example), or on the TEM grid as residuals from the sample preparation.

In order to minimize contamination, the samples were shipped as follows: immediately after the optical measurements, the sample grids were placed in a standard TEM grid holder, and the holder was encapsulated in polypropylene centrifuge tubes filled with nitrogen, and rigorously sealed with parafilm. The tubes were shipped from Cardiff to Antwerp in room-temperature packaging via next-day delivery. The samples were then measured within two days of arrival, and opened immediately before loading onto the electron microscope for imaging.

Notably, in a first round of experiments (not included in the results presented in the main text), the SiO_2 film of the TEM grid was cleaned and activated by the standard Piranha solution with reduced sulfuric acid (5%). The grid was incubated for 1 hour at 55 °C in 10 mL etching solution of 500 µl H_2SO_4 (99%) diluted with 9.5 mL of 30% H_2O_2.

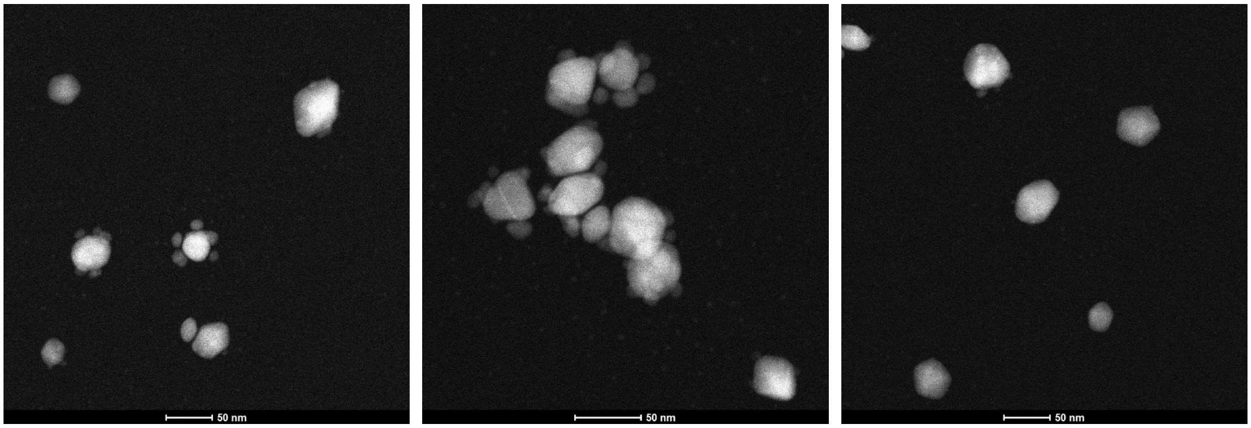

Figure S3. High-angle annular dark-field scanning TEM (HAADF-STEM) images of Ag particles on the SiO_2 windows which were cleaned and activated with a protocol involving sulfuric acid in the first round of experiments, after optical characterisation. For most particles, a set of smaller surrounding debris is visible.

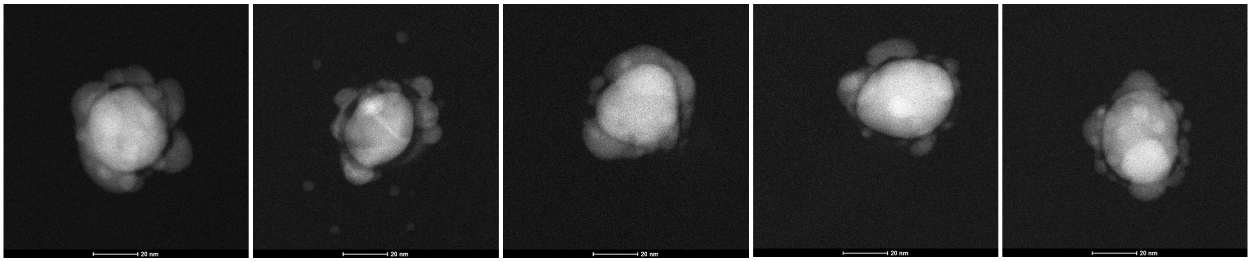

Figure S4. Same as Fig. S3, but zooms on selected Ag particles whose cross-section spectra showed a plasmon resonance peak.

While plasmonic NPs were identified in the optical measurements, subsequent electron microscopy indicated that many NPs in this batch were either completely converted to or were surrounded by debris containing sulfur (most likely Ag_2S) as shown in Fig. S3. These also included NPs whose optical cross-section displayed plasmonic peaks in the preceding measurements(see Fig. S4). Energy-dispersive X-ray (EDX) spectroscopy, shown in Fig. S5, confirmed the sulfur content of the debris, revealed by a characteristic sulfur peak emerging at 2.5 keV and the corresponding decrease of the Ag peaks at 0.3 and 3.0 keV. EDX maps such as the one displayed in Fig. S5 top right were acquired using the Super-X detector of the Tecnai Osiris TEM operated at 200 kV. The maps were generally acquired for 10 min at a current of 150 pA.

These findings in the first round of experiments indicated that the piranha solution might leave some H_2SO_4 on the grid, which might dissolve in anisole. After optical imaging the sample grid was held by a reverse-action tweezer and air-dried at 32°C, which could allow the residual to deposit on the grid surface and the particles, promoting sulfidisation.

In the second round of experiments, the sulfuric acid in the piranha solution was substituted with hydrochloric acid (see the Sec. 2 of the main paper). We found that this change in the protocol still granted NP immobilization, but avoided the formation of the obvious debris around particles. Since there was no structure observed in HAADF-STEM indicating a surface layer such as Ag_2S in the second round, EDX measurements were done only on a few particles. An example for particle #14 is shown in Fig. S6. The summed spectrum of the whole area contains no clear S peak, and the S map shows only uncompensated background signal. These results show, that within the limited signal-to-noise ratio of EDX, no S could be detected. Compared to the first round of experiments (Figs. S3 - S5), a possible Ag_2S surface layer must be very thin. As the grid is made of silica, the oxygen signal does not allow to locate a possible Ag_2O layer.

It should be noted that due to the low signal-to-noise ratio in EDX measurements, detecting thin (0.3–1.6 nm) sulfide layers as introduced in the main text is a challenge. Achieving sufficient sensitivity in EDX maps requires long acquisition times at higher beam currents than HAADF-STEM imaging, which induces beam damage. As a result, the spatial distribution of the element maps would be questionable, not only due to particle reshaping but also due to the possibility that the chemical modification could have occurred during the EDX measurements by material released from the support.

Regarding the possible origin of the sulfur, we revisited the protocol used. A thinkable origin could be the stainless steel reverse-action tweezers used to hold the grid during the cleaning and grid functionalisation process. They might contain small amounts of iron sulfide, which could react with the etchant used in a reaction $FeS + 2\,HCl \rightarrow FeCl_2 + H_2S$. The silica surface on the grid was functionalized with reactive amine groups which might react with the H_2S [S3] and carry the sulfide to the next step. The sample Ag NPs were in contact with the silica + amine surface in both polar and apolar solvent, and also under light irradiation during the measurement, which can be important for the reactivity of silver. Oxidation of silver can also be promoted by UV light. Generally speaking, it is well known that tarnish commonly forms on silverware when left in atmosphere for extended periods of time. This by itself indicates that the atmospheric sulfur

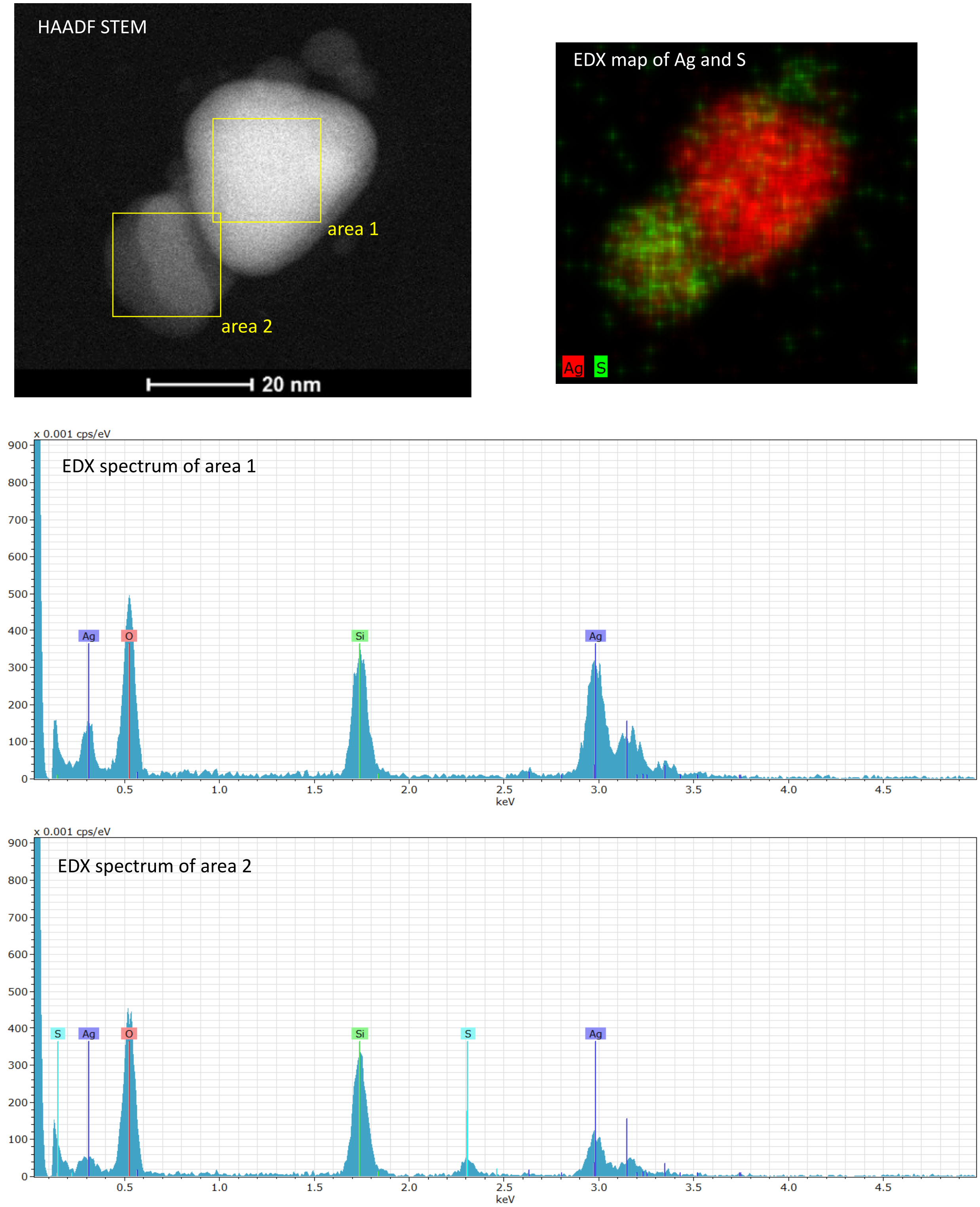

Figure S5. EDX elemental analysis. Top left: HAADF-STEM image of Ag particles as in Fig. S3. Two areas are indicated, whose EDX spectra are shown on the bottom. Top right: EDX map (smoothed with 3 pixels width) of the Ag peak net counts (red, 2.9 – 3.1 keV) and the S peak net counts (green, 2.2 – 2.4 keV).

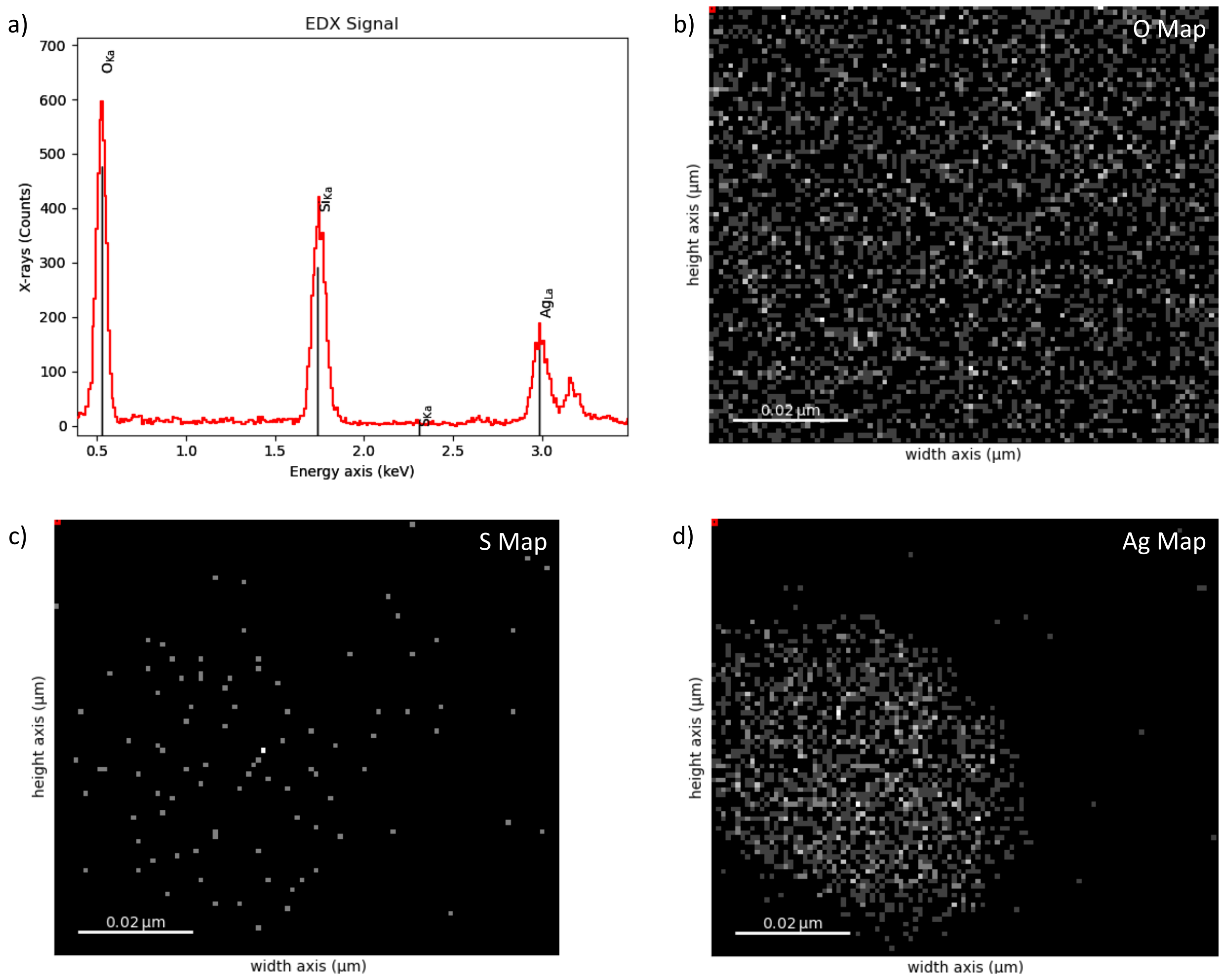

Figure S6. EDX elemental analysis of particle #14. a) EDX spectrum. EDX maps of b) the Ag peak counts (2.9 – 3.1 keV) c) the S peak counts (2.2 – 2.4 keV), and d) the O peak counts (0.4 – 0.6 keV).

or oxygen content is enough to promote tarnishing of exposed silver surfaces, with no need of being fostered by reactions specific to our protocol. Even more so in the case of silver in NP form, whose reactivity is increased by the high surface-to-volume ratio [S4, S5].

S.III. CORRECTION FOR FINITE REGION OF DETECTION

In our micro-spectroscopy experiments the imaged area is delimited along the dispersive direction by the input slit of the spectrometer having a width of 80 µm. The slit is imaged with same size onto the sensor, where it is matched along the orthogonal direction by the on-chip binning (we read out a bin of 5 pixels of 16 µm pitch). Considering the magnification of about 80× from sample to sensor (characterized experimentally by a controlled displacement of the sample stage), the $80 \times 80\,\mu\text{m}^2$ region of interest on the sensor corresponds to a square imaged area of lateral size $L = 1.0\,\mu\text{m}$ on the sample. This value was chosen to accommodate a particle image – which for sufficiently small particles corresponds to the point spread function (PSF) of the imaging system – while leaving some margin for possible lateral drifts over the acquisition time (few tens of seconds; the typical thermal drift of the imaged position is about 100 nm/min).

However, the mathematical PSF of a point source extends infinitely in space, albeit in practice only few rings (if any) are typically visible above the background noise level. This means that only a fraction $f < 1$ of the particle signal is detected as the tails of the PSF are cropped by the spatial filtering of the image. Note that $f^{\mathrm{BF}} \neq f^{\mathrm{DF}}$, since the PSF in brightfield (BF) and darkfield (DF) images differ due to the different angular range of excitation and the different contrast mechanism. Specifically, in DF, the scattered intensity is measured, with a PSF determined by the objective numerical aperture (NA) and particle focus (within an approximated scalar diffraction theory neglecting the polarization dependence) whereas in BF, the transmitted power is measured, which results from the interference between incident and scattered field, leading to a partially coherent imaging. Matching condenser and objective NA, as we do in our experiment, the PSF in BF is of similar size as the one in DF.

In our quantitative analysis, the reduction of the excitation and scattering signal due to the finite area of detection is accounted for by rescaling σ_{ext} and σ_{sca} by f^{BF} and f^{DF}, respectively. We determine f^{BF} and f^{DF} for our set-up with the following procedure. Widefield images are acquired with a low-noise scientific sCMOS camera (PCO Edge 5.5). Illumination is provided by a 100 W halogen lamp (Nikon V2-A LL 100 W), filtered using bandpass filters (Thorlabs FKB-VIS-40) with centre wavelengths of (450, 500, 550 and 600) nm, so to address the wavelength dependence of f. The illumination and detection NA ranges for BF and DF are the same as in the experiment (namely, we use the same condenser and set of 3D printed apertures, and the same objective) to ensure we characterize the same PSF. We also use the same silver nanohedra sample, although ideally the PSF is the same for any isotropic subwavelength object.

We analysed the acquired transmission and scattering images using *Extinction Suite*, a plug-in for the image processing programme ImageJ which we have been developing within our group – see `https://langsrv.astro.cf.ac.uk/Crosssection/Crosssection.html` and publications referenced therein. An analysis routine determines the particle position via a Gaussian fit of its transmission or scattering image; the extinction or scattering magnitude is quantified by integrating over a circular region of interest of radius R_{i} centred around the particle position. Fig. S7a shows a measurement of the extinction as a function of R_{i}, after

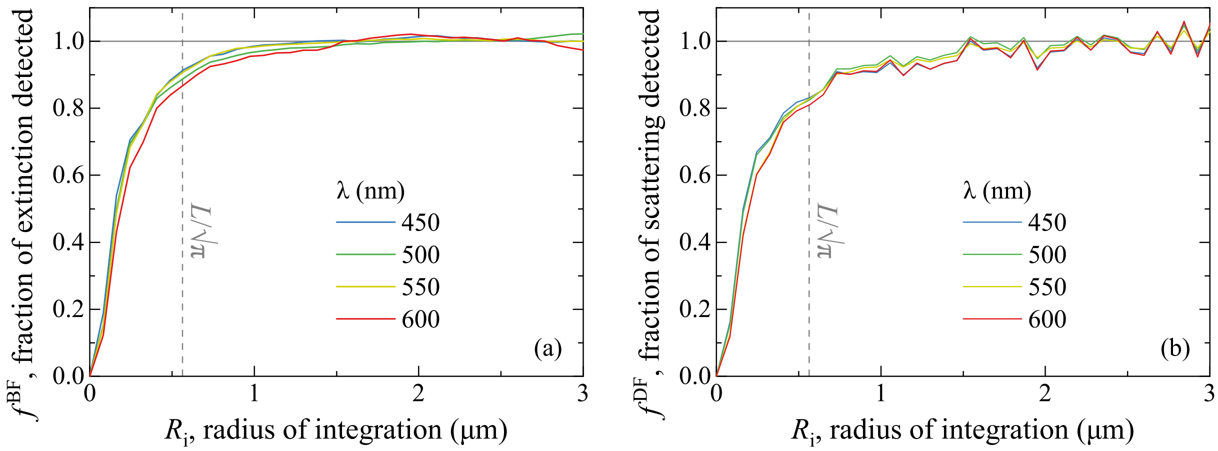

Figure S7. Fraction of (a) extinction and (b) scattering detected in imaging as a function of the integration radius R_i, normalized to its saturation value at large R_i indicated by the horizontal guideline at $f = 1$. The vertical dashed lines correspond to the equivalent radius of the imaged sample region in our microspectroscopy experiments.

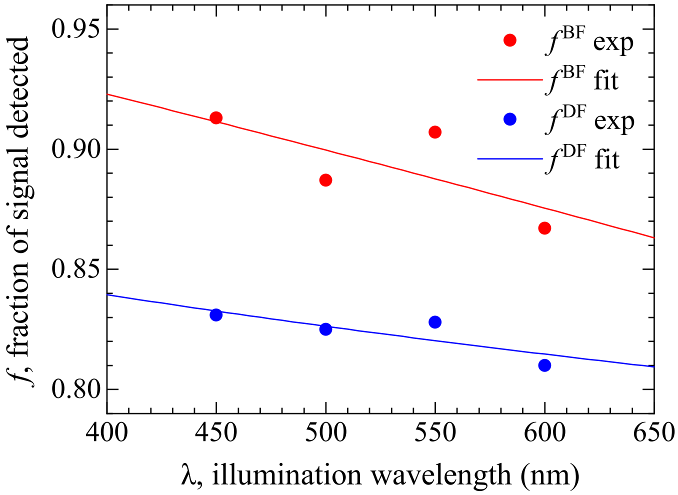

Figure S8. Measured values of f^{BF} and f^{DF} in four colour channels (circles). The wavelength dependence is fitted by a power law (lines) given by Eq. (S1).

subtraction of the local background measured over an area πR_i^2. The extinction saturates at about $R_i = 3\lambda/\mathrm{NA} \simeq 1.7\,\mu\mathrm{m}$, above which fluctuations of the local background dominate. The scattering magnitude shown in Fig. S7b exhibits a similar behaviour with a slightly slower saturation (at about 2 μm). We associate the value $f = 1$ to the saturation magnitude – indicated by the horizontal lines in Fig. S7a,b – and normalize to it the extinction or scattering.

We estimate f^{BF} and f^{DF} in our micro-spectroscopy experiments at the equivalent radius $L/\sqrt{\pi} = 564\,\mathrm{nm}$ (vertical dashed line) which has the same area as the square region detected in micro-spectroscopy; the resulting values are reported in Fig. S8 for the four colour channels used.

The experimental data are fitted with the phenomenological function

$$f(\lambda) = 1 - (\lambda/A)^p \tag{S1}$$

and the parameters ($A = 4091\,\text{nm}$, $p = 1.18$) for BF and ($A = 71\,908\,\text{nm}$, $p = 0.353$) for DF. The functions $f^{\text{BF}}(\lambda)$ and $f^{\text{DF}}(\lambda)$ are used to correct the measured cross section magnitudes according to Eq. (S2) below. The decreasing trend for longer λ observed in in Fig. S8 is explained by the scaling of the PSF with λ; for instance, the Airy function (which describes the focal spot created by a perfect lens with a circular aperture in the paraxial approximation) has a first dark ring of diameter $1.22\lambda/\text{NA}$. Note that this scaling is consistent with the limit behaviour $f \longrightarrow 1$ for $\lambda \longrightarrow 0$ of the fitting function of Eq. (S1). We estimate that the error in the determined factors is about 5 to 10%, mostly due to the determination of the saturation value for large R_{i}, which is affected by fluctuations in the background value that increase for larger integration areas.

S.IV. CHARACTERIZATION OF THE BF-TO-DF ILLUMINATION INTENSITY

Another crucial parameter of the experimental set-up for quantifying the cross-section magnitude is the BF-to-DF ratio of the illumination intensity. The need for this parameter arises because in DF the excitation intensity cannot be directly measured, and it has therefore to be retrieved from the BF background through a proportionality factor. We call this parameter ξ, and it acts as a scaling factor for the magnitude of $\sigma_{\rm sca}^{\rm DF}$ – see Eq. (S2) below. ξ is governed by the amount of light blocked by the BF and DF apertures in the back focal plane (BFP). It is therefore possible to derive a simple analytical expression of ξ (Eq. (3) in Ref. [S6]) assuming an aplanatic behaviour of the condenser lens. However, in most microscopy set-ups the illumination is not homogeneous over the BFP of the condenser; moreover the condenser transmittance drops towards the edges of is aperture. These effects add up to give a strong decrease of the illumination intensity at large NA values, which effectively lower the DF illumination. Such angular efficiency of the excitation path can be characterized experimentally and used to correct ξ as described in § S.VI.B in Ref. [S7]

In this work (similar to what we did already in Ref. [S6] for the polystyrene beads) we have instead measured $\xi = 2.04$ directly for the specific BF and DF 3D-printed apertures used in the experiment with the following procedure. Using an excitation path replicating the microspectroscopy experiments, a 1.45 NA objective is used in the detection path to collect all exciting light also in the DF illumination configuration, which has a maximum of 1.34 NA. A clean glass slide is used in place of the sample (to hold the immersion oil of objective and condenser) and Köhler illumination is adjusted focussing the field aperture. To minimize the effect of chromatic aberrations and reproduce the experimental focussing conditions a colour filter centred at 550 nm and a width of 40 nm (Thorlabs FBH550-40) is used, which is the spectral region of the plasmonic resonance of the decahedra. Widefield images are then acquired with a scientific sCMOS camera (PCO Edge 5.5) using the BF and DF 3D-printed apertures. The illumination intensity is proportional to the mean value of the camera readout over a region of interest in the centre of the field of view. Taking the ratio of BF to DF readout (after subtracting from both the dark offset of the camera digitizer) yields ξ. Note that this procedure neglects the angular dependence of the collection efficiency; however, for the high-quality objective used (Nikon MRD01905, 100× 1.45 NA PlanApo Lambda series) we expect this dependence to be weak over the range up to 1.34 NA of the transmitted illumination, considering the significant margin to the objective NA.

S.V. SPECTROSCOPY OF ALL PARTICLES

Let us report here for convenience the formulas derived in Ref. [S6] that we use to quantify the cross section magnitude, slightly adapted to match the notation of this work

$$\sigma_{\text{sca}}^{\text{DF}} = \frac{L^2}{f^{\text{DF}}} \frac{\xi}{\eta^{\text{DF}}} \frac{S_{\text{NP}}^{\text{DF}} - S_{\text{bg}}^{\text{DF}}}{S_{\text{bg}}^{\text{BF}}} \quad \text{(S2s)} \qquad \sigma_{\text{abs}}^{\text{BF}} = \frac{L^2}{f^{\text{BF}}} \frac{S_{\text{bg}}^{\text{BF}} - S_{\text{NP}}^{\text{BF}}}{S_{\text{bg}}^{\text{BF}}} - (1 - \eta^{\text{BF}}) \frac{\zeta}{\xi} \sigma_{\text{sca}}^{\text{DF}} \quad \text{(S2a)}$$

where $S(\lambda)$ are the the scattering/extinction spectra detected (after subtraction of the dark offset of the CCD digitizer) under DF/BF illumination, imaging either the nanoparticle (NP subscript) or the background (bg subscript) in an empty area nearby. The parameters f, L, and ξ are specific to the experimental set-up and settings only (they have the same value for all measured particles) and were discussed in Sec. S.III and Sec. S.IV. Conversely, the parameters ζ and η are specific to each particle and, as mentioned in the article, encode the directional properties of scattering with respect to excitation and detection, respectively.

Fig. 2 of the article shows the measured and simulated quantitative optical cross-section spectra $\sigma(\lambda)$ of six representative particles. The data for all twenty particles investigated in this work are shown in Fig. S9–S12; a transmission electron microscopy (TEM) tomographic reconstruction of each particle is included as an inset. The study we presented in the article focuses on $\sigma_{\text{sca}}^{\text{DF}}(\lambda)$ which dominates the response of the investigated particles. The untreated absorption spectra (not shown here) display for most particles negative values over a large spectral range in correspondence of the dipolar plasmonic resonance governing the scattering spectra. To pinpoint the origin of such non-physical result (which would imply a net power emission from the particle) it is useful to look at the structure of Eq. (S2a). The first term is the measured $\sigma_{\text{ext}}^{\text{BF}}$ and the second term is the portion of BF scattering not collected by the objective, which is subtracted from the total extinction to isolate the absorptive contribution. Therefore $\sigma_{\text{abs}}^{\text{BF}} < 0$ can result from either underestimating $\sigma_{\text{ext}}^{\text{BF}}$ or overestimating $\sigma_{\text{sca}}^{\text{BF}} = (\zeta/\xi)\sigma_{\text{sca}}^{\text{DF}}$. The first can be corrected by decreasing f^{BF} and the latter by increasing f^{DF} or decreasing ζ. Each correction corresponds to different aspects of uncertainty in the experiment; specifically, f refers to spatial filtering (*e. g.* the particle drifts away from the centre of the imaged area during the acquisition, and hence f is lowered) and ζ to directional filtering (*e. g.* accuracy of the fabrication and positioning of the 3D printed apertures in the BFP of the condenser). For the spectra shown in Fig. S9–S12 we decreased the parameter f^{BF} by 20% to avoid negative values of the spectral average of the experimental absorption. Note that for these particles the opposite contributions of extinction and scattering have similar magnitude, so that they approximately balance each other; this implies that a similar result can be obtained by a 20% correction of either f^{DF} or ζ. An even more accurate measurement of the parameters for the experimental setup seems required to avoid such an adjustment. We emphasize that the same correction was used for all spectra shown.

Let us now turn our attention to the scattering parameters, $\zeta(\lambda)$ and $\eta(\lambda)$, which are computed for each particle and correlated to $\sigma(\lambda)$ in Fig. S9–S12 (bottom panels). It is instructive to compare

these numerical simulations – which take into account the complexity of the particle shape – to our previous analytical calculations performed in the electrostatic approximation – see section S.V. of Ref. [S6]. Let us start with η, which is the fraction of the scattering power collected by the objective. Given the excitation and detection NA ranges of the experiment and assuming a homogeneous immersion medium, one finds $\eta^{\mathrm{BF}} = \eta^{\mathrm{DF}} = 0.148$ for a polarisability perpendicular to the optical axis and $\eta^{\mathrm{BF}} = 0.136$, $\eta^{\mathrm{DF}} = 0.111$ for an isotropic polarisability. The simulated η compares well with this estimate, being closer to the spherical value for most particles. Note that η is determined by the orientations of the electric dipoles excited in the particle: the larger the angle formed with the optical axis of the objective, the lower the fraction of emission collected, resulting in the largest η for a dipole lying flat on the substrate. Looking at the simulations below, these considerations also explain why $\eta^{\mathrm{BF}} > \eta^{\mathrm{DF}}$ – as more inclined dipoles are excited in DF – and why for most particle η decreases for $\lambda < 450$ nm, where multipolar resonances are excited.

As for ζ, which is the BF-to-DF ratio of the total scattered power, no straightforward comparison to the values in the dipole limit ($\zeta = 1.22$ for an in-plane polarisability and $\zeta = 0.897$ for an isotropic polarisability) can be made. This is because in this set of measurements the NA range of the DF illumination reaches the edge of the condenser aperture (1.34 NA) where the illumination intensity is significantly reduced – see Fig. S7 of Ref. [S7]. The numerical modelling used in this work takes into account the angular dependence of the illumination intensity based on our experimental characterization of the performance of the condenser – see Ref. [S7]. Conversely, the analytical calculations assume a homogeneous filling of the back aperture of the condenser, thereby overestimating the DF illumination and scattered power, which leads to a lower ζ. Along the same line of reasoning, the dip of ζ for $\lambda < 450$ nm implies that the multipolar modes in that region are comparatively better excited by the tilted illumination of DF.

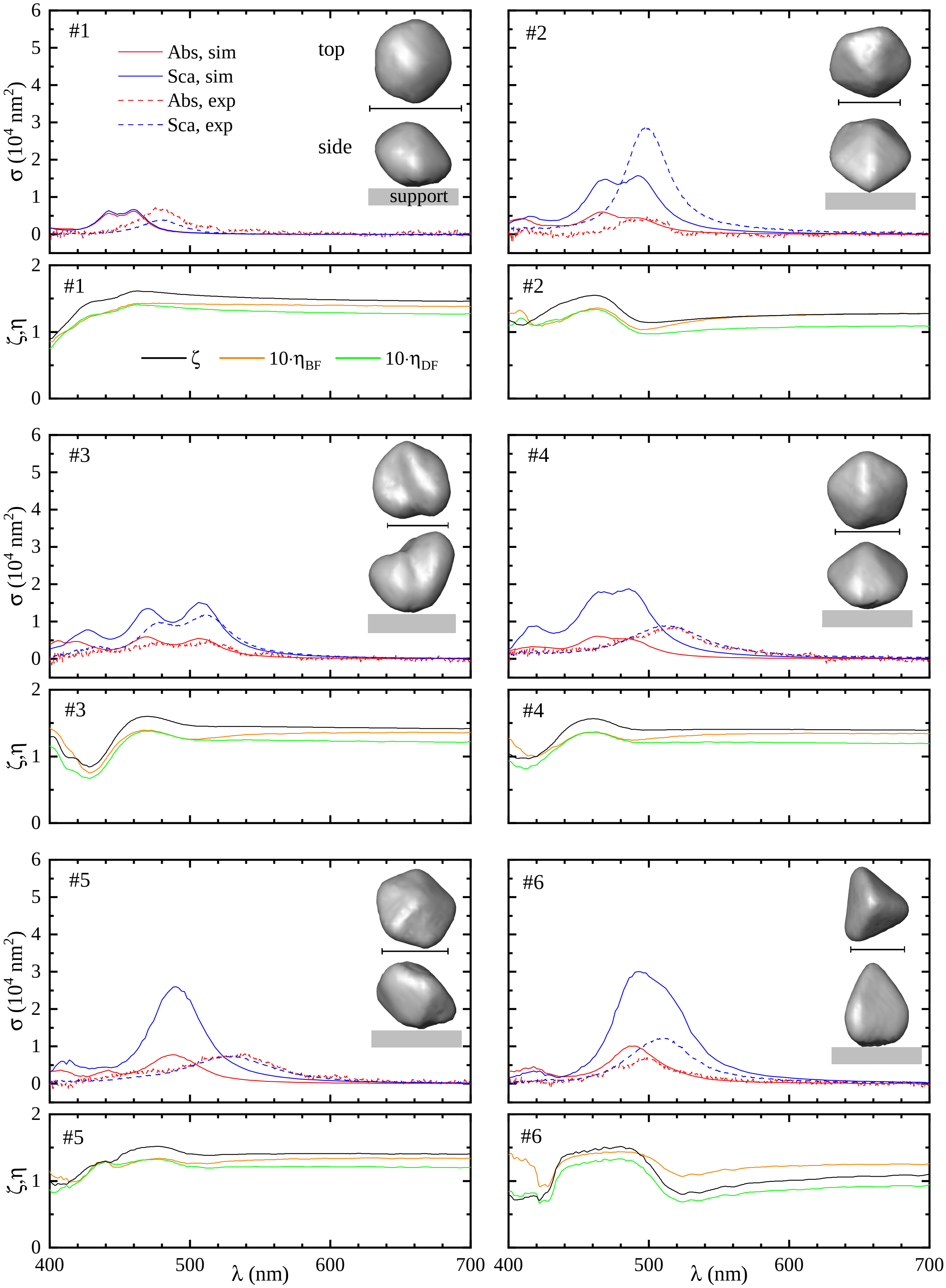

Figure S9. Measured and simulated optical cross section spectra (top) and parameters ζ and η (bottom) of the particles #1 to #6. The insets are tomographic reconstructions (scale bar is 40 nm).

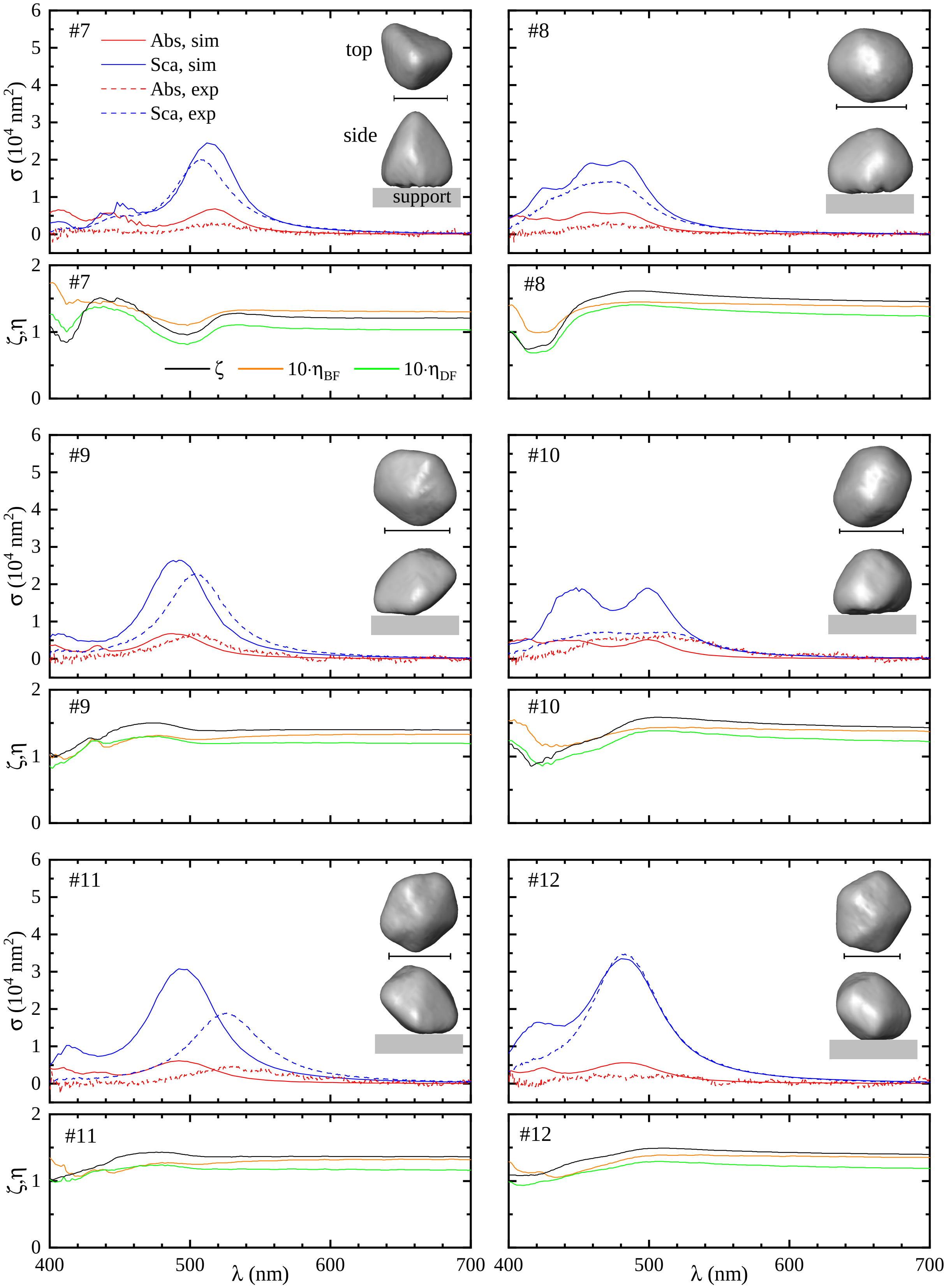

Figure S10. Same as Fig. S9, but for particles #7 to #12.

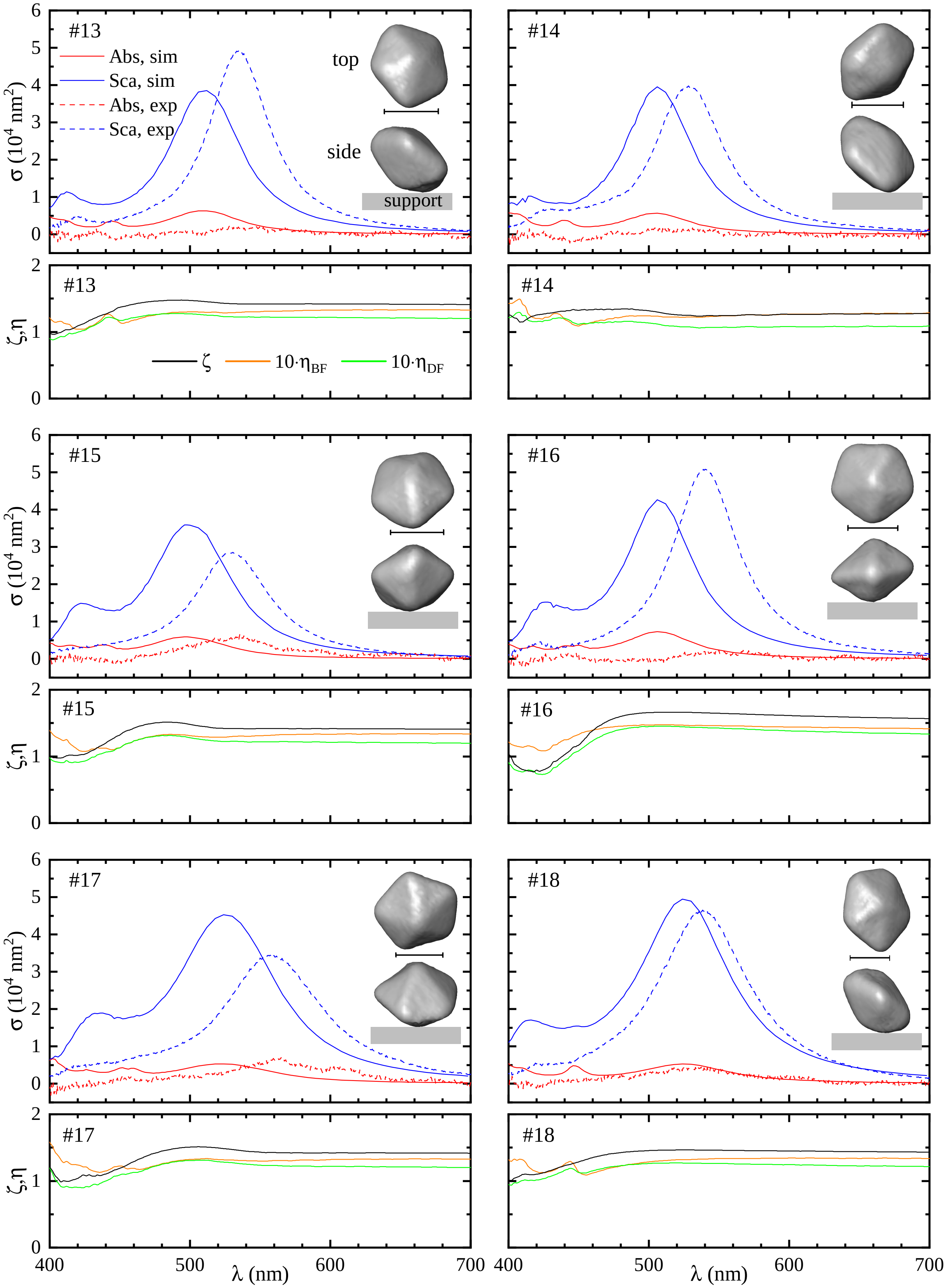

Figure S11. Same as Fig. S9, but for particles #13 to #18.

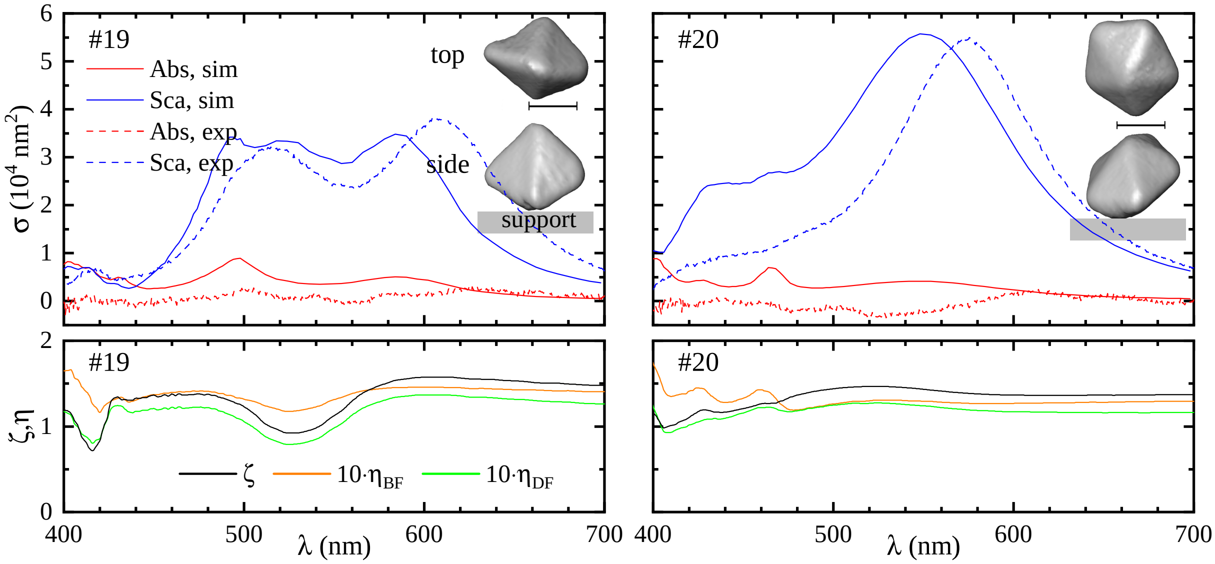

Figure S12. Same as Fig. S9, but for particles #19 and #20.

S.VI. MORPHOLOGY RECONSTRUCTION FROM HAADF-STEM TOMOGRAPHY AND ITS INFLUENCE ON THE SIMULATED CROSS-SECTIONS

A. Remeshing

As described in the main text, we investigated the dependence of our results on the geometry reconstruction method, and evaluated three procedures (R1 to R3). The different reconstruction methods were applied to a selection of particles, and the resulting volumes, and volume-to-surface ratios are given in Table S1 along with a summary of the parameters used for each algorithm. Generally we find that the volume vary by some 5% to 10%, with R1 resulting in the lower and R2 in the higher volumes. The volume to surface ratios also vary by some 5% to 10%, but there is no clear trend across the particles for the different reconstructions.

While with the R1 procedure the resulting mesh can be directly imported and re-meshed by Comsol, R2 and R3 sizeably increase the number of surface elements defining the particle, which could not be imported, processed, and meshed reliably with Comsol. We therefore reduced the number of surface elements using the free software Meshlab and a procedure illustrated in Fig. S13 for two exemplary particles of different appearance. First the number of faces was changed to 1000 using the option ‘quadratic edge collapse’, then the result was turned into pure triangular mesh, and finally the errors in the geometry (such as holes or crossing mesh elements) were repaired by the option ‘remove non manifold edges by removing faces’. The resulting mesh was then imported into Comsol.

Table S1. Volume and volume-to-surface ratio of particles reconstructed with different procedures. The parameters identifying the reconstruction procedures R1, R2, R3 are given with the following abbreviations: It: iterations, N: factor of downsampling, Sm: smoothing, Rm: remeshing.

NP #	R1		R2		R3	
	It = 15, N = 12, Sm = No, Rm = No		It = 15, N = 4, Sm =Yes, Rm = Yes		It = 100, N = 1, Sm = Yes, Rm = Yes	
	V (10^4 nm^3)	V/S (nm)	V (10^4 nm^3)	V/S (nm)	V (10^4 nm^3)	V/S (nm)
3	4.09	6.65	4.29	6.79	4.10	6.50
6	4.67	5.88	5.24	5.19	5.20	7.07
7	4.92	6.87	5.42	7.24	5.33	7.12
18	12.2	9.53	12.3	9.53	11.9	9.30
19	12.6	9.13	14.6	9.80	14.0	9.46
20	18.4	10.8	18.2	10.6	17.4	10.4

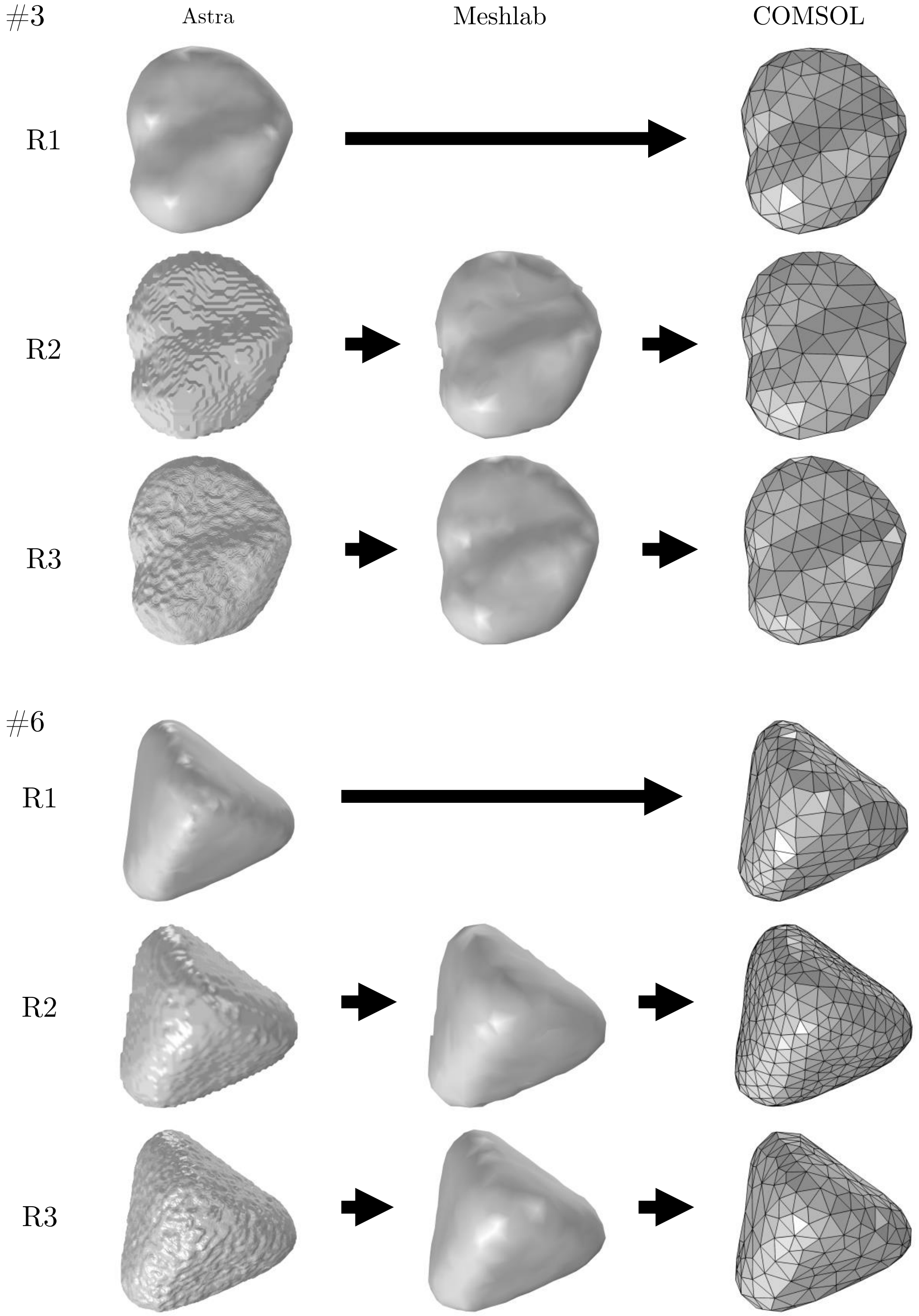

Figure S13. Comparison of three geometry reconstruction procedures (R1 to R3) for two different particles (#3 and #6) viewed from the top. For R1 the 3D reconstruction is directly imported into Comsol and then meshed, while for R2 and R3 an intermediate step is introduced to reduce the number of faces defining the geometry.

B. Cross-section spectra

Fig. S14 shows the scattering cross-section spectra of the six particles studied in the article obtained using the different TEM tomography reconstruction procedures R1 to R3. There is no clear common trend of the effect of the different reconstructions. We typically see variations of some 10 to 20 nm in peak position, and in peak splitting, and some 5% to 20% in peak amplitude.

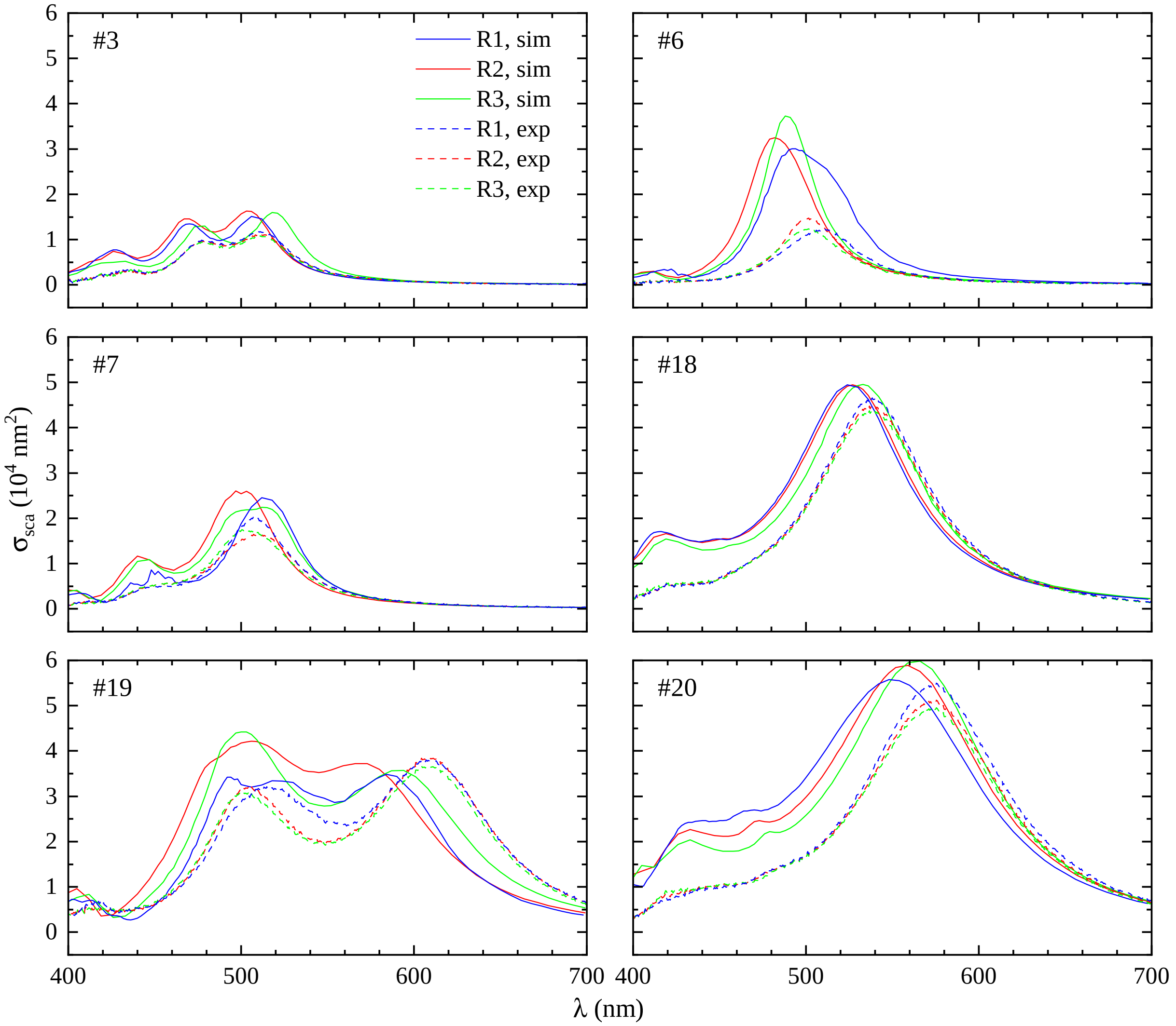

Figure S14. Simulated (solid lines) and experimental (dashed lines) scattering cross section spectra of the particles #3, #6, #7, #10, #18, #19, and #20, as labelled, for different geometry reconstruction procedures R1 (blue lines), R2 (red lines), and R3 (green lines).

For particle #3 and #6 COMSOL could process the R2 geometries without remeshing, therefore we can use these two particles to investigate the effects of the remeshing. In Fig. S15 we show the simulated cross section spectra for these two particles. We find a small blue-shift of approximately 5 nm due to the remeshing for both particles, and an increase in amplitude below 1 %. The effects should be even smaller for larger particles, which are less sensitive for small surface changes that are caused by the meshing. All other simulations shown in the supplement or in the article uses the outlined remeshing steps for R2 and R3, and the 'rm' label is dropped.

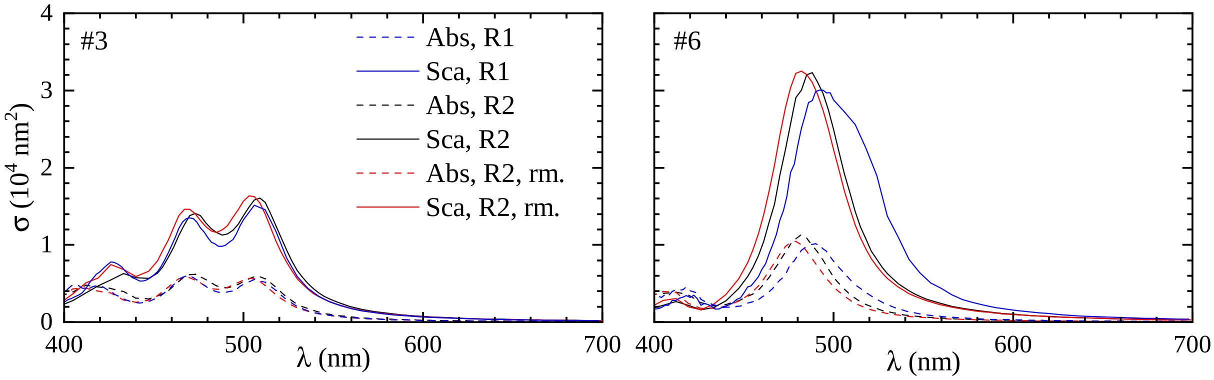

Figure S15. Simulated cross-section spectra of particles constructed via the R2 algorithm, with (labeled rm.) and without remeshing before importing into COMSOL.

C. Rotating three-dimensional renderings of the particle geometry

We provide as online material animations of the reconstructed geometries of all reported NPs.

S.VII. SURFACE AND INTERFACE DRUDE DAMPING

In this section we investigate the effect of increasing the Drude damping in the Ag permittivity, to model the increased surface or defect scattering in the particles compared to the permittivity datasets [S8] measured by ellipsometry on thin films. Such an increase is expected due to the particle size being smaller than the crystallite sizes in the measured films, and the additional crystal defects which can be created in the colloidal growth [S2, S9]. We fit the data set [S8] with the Drude model $\varepsilon(\omega,\gamma) = \varepsilon_\infty - \omega_p^2/(\omega^2 + i\omega\gamma)$ in the range 400 to 700 nm. The fit parameters are $\varepsilon_\infty = 3.8575$, $\omega_p = 1.3666 \times 10^{16}\,\mathrm{s}^{-1}$ and $\gamma = 7.7849 \times 10^{13}\,\mathrm{s}^{-1}$. The resulting analytical permittivity is shown in Fig. S16 along with the fitted experimental dataset $\varepsilon_{\mathrm{exp}}(\omega)$ of Ref. [S8]. We note from panel b that the imaginary part of the permittivity has some deviations from the Drude model in this range, and it has been shown [S10] that additional poles are needed for an accurate fit. However, since we are here only interested in modelling the change of the permittivity by increasing Drude damping, the simpler model suffices. Following [S11], we then add a damping $\Delta\gamma = g v_F/R$ where $v_F = 1.36\times10^6$ m/s is the Fermi velocity, and the equivalent radius R is calculated from the volume based on a spherical particle, $R = \sqrt[3]{3V/4\pi}$, and replace γ with $\overline{\gamma} = \gamma + \Delta\gamma$ in the modified permittivity $\varepsilon(\omega,\overline{\gamma}) = \varepsilon_\infty - \omega_p^2/(\omega^2 + i\omega\overline{\gamma})$. We vary the damping parameter g, and the resulting change to the imaginary part of the permittivity $\varepsilon(\omega,\overline{\gamma})$ is shown in Fig. S16b,c. For particle #20 the permittivity changes less compared to #3 due to its larger R. The real part of the permittivity is changed by less than 0.1% therefore this is neglected here. We take the change of the Drude permittivity $\Delta\varepsilon(\omega,\overline{\gamma}) = \varepsilon(\omega,\overline{\gamma}) - \varepsilon(\omega,\gamma)$, and add it to the measured data $\varepsilon_{\mathrm{exp}}(\omega)$, resulting in the modified permittivity $\varepsilon_m(\omega,\overline{\gamma}) = \varepsilon_{\mathrm{exp}}(\omega) + \Delta\varepsilon(\omega,\overline{\gamma})$ used in the simulation.

In the article we show in Fig. 4 the effect of the permittivity change on the scattering cross section, while Fig. S17 shows the effect on the absorption cross section. The simulated absorption increases for stronger damping as expected. The experimental absorption, like already seen for

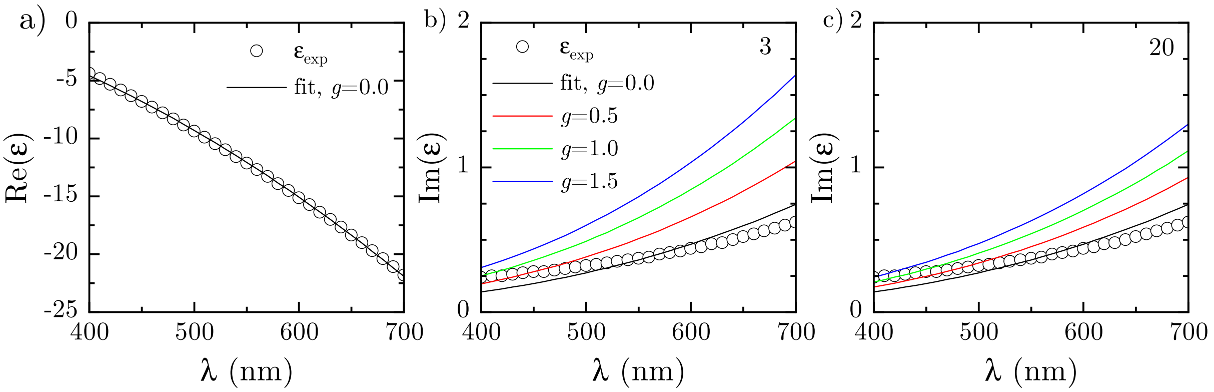

Figure S16. Fit of the experimental permittivity dataset of S8 with the Drude model and additional damping. a) Real part, data (circles), and model (line). b) Imaginary part, data (circles), and model (lines) for $g = 0$ (black), as well as with the added damping using $g = 0.5$ (red), 1.0 (green), and 1.5 (blue), for particle #3. c) Same as b) but for particle #20.

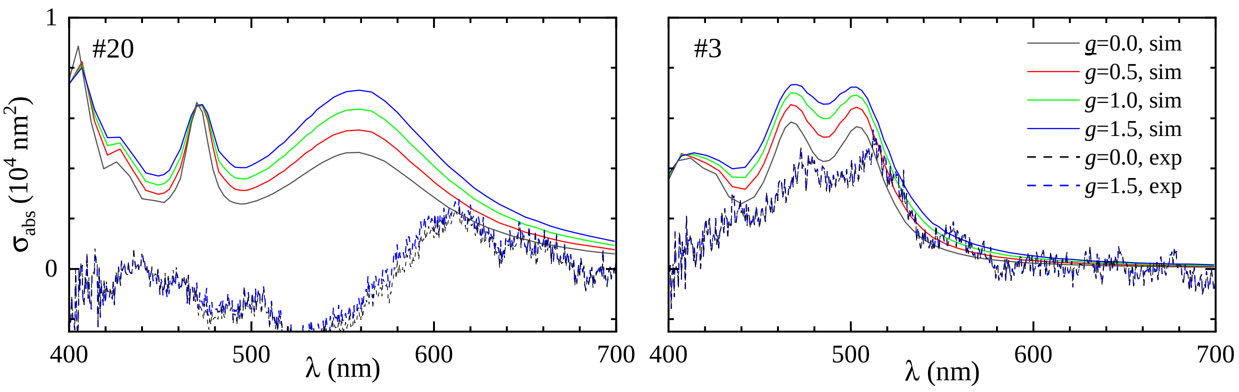

Figure S17. Simulated (solid lines) and measured (dashed lines) absorption cross section spectrum of particle #20 (left) and #3 (right) for different magnitudes g of the surface damping.

the scattering, is unaffected because the simulated parameters η and ζ are only weakly affected by the increased damping (in the electrostatic limit, they are dispersionless and depend only on the particle geometry but not its material properties.).

S.VIII. CROSS-SECTION SPECTRA FOR SULFIDE OR OXIDE TARNISHING

We have already discussed in Sec. S.II that the chemical composition of the tarnish layer on the NP surface is uncertain, although silver sulfide (Ag_2S) seems the most likely candidate based on previous reports in literature. In this section, we investigate a possible different composition of such layer, namely silver oxide (Ag_2O), comparing the simulated spectra to those obtained with an Ag_2S layer, which was used in the main text for the NPs #3 and #20 – see Fig. 6.

The permittivity spectra used as material properties were taken from Ref. [S12] for Ag_2S as in the main text, and from Ref. [S13] for Ag_2O. To compare the effect of the two materials, it is sufficient to evaluate the cross-section spectra for normal incidence illumination, given that the cross-section spectra quantitatively simulating the measurements, which use a range of illumination directions, are reported for Ag_2S tarnish layers in the main text. The layers are modelled as described in Sec. 3.3.

The resulting cross-section spectra are shown in Fig. S18. For the Ag_2S tarnish layer, the same thicknesses as in the main text are used, while for the Ag_2O tarnish layer, the thicknesses are chosen to provide a similar change of the cross-sections as for the Ag_2S tarnish layer. For particle #20, the scaling factor for the Ag_2S layer is 0.97, yielding a thickness of approximately 1 nm. The layer redshifts the dipolar peak and decreases its amplitude, as discussed in the main text. For the Ag_2O layer, a scaling factor of 0.95 was used, yielding a thickness of about 1.6 nm. We find that the Ag_2O layer results in a slightly larger amplitude reduction for a given shift. This would slightly increase the deviation from the experiment seen in Fig. 6 for the Ag_2S layer. For particle #3 , the scaling factor for the Ag_2S layer is 0.985, yielding a thickness of approximately 0.3 nm. For the Ag_2O layer, a scaling factor of 0.98 was used, yielding a thickness of about 0.4 nm. Here the lower

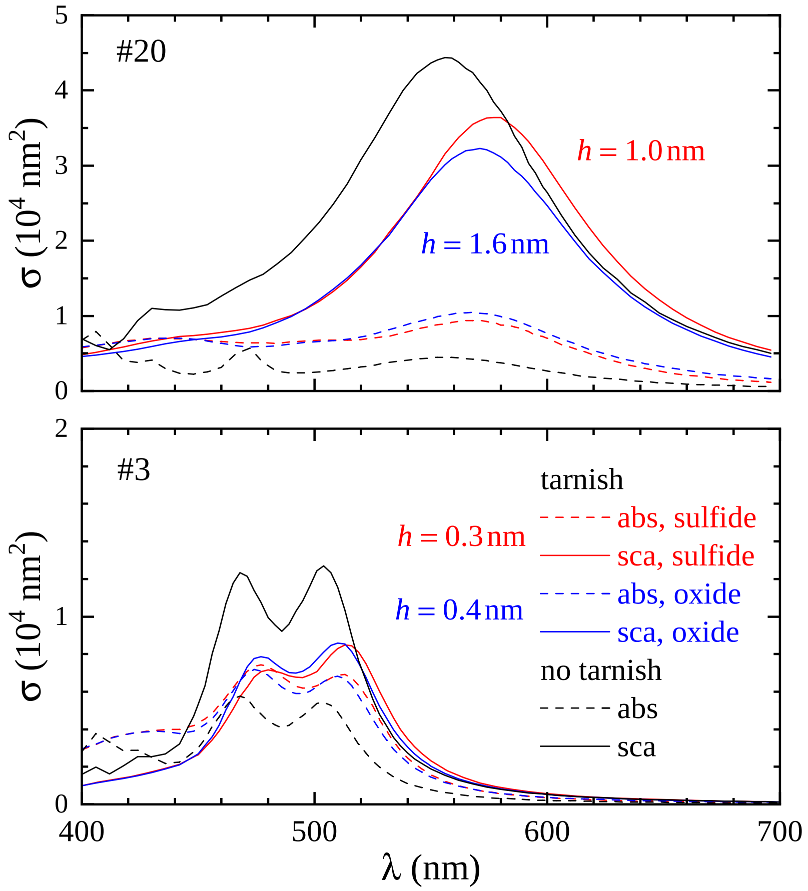

Figure S18. Simulated cross-section spectra of particle #20 (top) and #3 (bottom) for a silver-sulfide (red) and a silver-oxide (blue) tarnish layer of thickness *h* as given covering the particle, for normal incidence illumination.

shift for the oxide layer would slightly decrease the deviation from the experiment. The actual morphology of the tarnish is likely more complex than the thin layer of homogeneous thickness used here for modelling; for example one could expect a higher reactivity of corners. Therefore, the observed small differences between Ag_2S and Ag_2O layers are not conclusive. Notably, Ag_2O could not be detected in the EDX results (see Sec. S.II) due to the presence of oxygen in the SiO_2 support, so that even the Ag_2O thickness of 1.6 nm used for particle #20 would not be easily visible in EDX.

[S1] B. Pietrobon and V. Kitaev, Photochemical synthesis of monodisperse size-controlled silver decahedral nanoparticles and their remarkable optical properties, Chem. Mater. **20**, 5186 (2008).

[S2] X. Zheng, X. Zhao, D. Guo, B. Tang, S. Xu, B. Zhao, W. Xu, and J. R. Lombardi, Photochemical formation of silver nanodecahedra: Structural selection by the excitation wavelength, Langmuir **25**, 3802 (2009).

[S3] C. N. Okonkwo, J. J. Lee, A. D. Vylder, Y. Chiang, J. W. Thybaut, and C. W. Jones, Selective removal of hydrogen sulfide from simulated biogas streams using sterically hindered amine adsorbents, Chem. Eng. J. **379**, 122349 (2020).

[S4] M. D. Mcmahon, R. Lopez, H. M. Meyer, L. C. Feldman, and R. F. Haglund, Rapid tarnishing of silver nanoparticles in ambient laboratory air, Appl. Phys. B **80**, 915 (2005).

[S5] J. L. Elechiguerra, L. Larios-Lopez, C. Liu, D. Garcia-Gutierrez, A. Camacho-Bragado, and M. J. Yacaman, Corrosion at the nanoscale: the case of silver nanowires and nanoparticles, Chem. Mater. **17**, 6042 (2005).

[S6] A. Zilli, W. Langbein, and P. Borri, Quantitative measurement of the optical cross sections of single nano-objects by correlative transmission and scattering microspectroscopy, ACS Photonics **6**, 2149 (2019).

[S7] Y. Wang, A. Zilli, Z. Sztranyovszky, W. Langbein, and P. Borri, Quantitative optical microspectroscopy, electron microscopy, and modelling of individual silver nanocubes reveal surface compositional changes at the nanoscale, Nanoscale Adv. **2**, 2485 (2020).

[S8] H. U. Yang, J. D'Archangel, M. L. Sundheimer, E. Tucker, G. D. Boreman, and M. B. Raschke, Optical dielectric function of silver, Phys. Rev. B **91**, 235137 (2015).

[S9] H. Xu and B. J. Wiley, The roles of citrate and defects in the anisotropic growth of Ag nanostructures, Chem. Mater. **33**, 8301 (2021).

[S10] H. S. Sehmi, W. Langbein, and E. A. Muljarov, Optimizing the Drude–Lorentz model for material permittivity: Method, program, and examples for gold, silver, and copper, Phys. Rev. B **95**, 115444 (2017).

[S11] C. Voisin, N. Del Fatti, D. Christofilos, and F. Vallée, Ultrafast electron dynamics and optical nonlinearities in metal nanoparticles, J. Phys. Chem. B **105**, 2264–2280 (2001).

[S12] J. M. Bennett, J. Stanford, and E. Ashley, Optical constants of silver sulfide tarnish films, J. Opt. Soc. Am. **60**, 224 (1970).

[S13] L. Pettersson and P. Snyder, Preparation and characterization of oxidized silver thin films, Thin Solid Films **270**, 69 (1995).
